# Working from Home, COVID-19, and Job Satisfaction

**DOI:** 10.1177/00197939241301704

**Published:** 2024-12-07

**Authors:** Inga Laß, Esperanza Vera-Toscano, Mark Wooden

**Affiliations:** * Inga Laß and Esperanza Vera-Toscano are Senior Research Fellows at the Melbourne Institute of Applied Economic and Social Research, University of Melbourne. Mark Wooden is Professor Emeritus at the Melbourne Institute of Applied Economic and Social Research, University of Melbourne. At the time this research commenced, Inga Laß was a Senior Researcher at the Federal Institute for Population Research (BiB) (Germany)

**Keywords:** telework, well-being, COVID-19 pandemic, HILDA Survey, gender, work–family balance

## Abstract

This article examines the impact of the growth in the incidence of working from home during the COVID-19 pandemic on workers’ job satisfaction. Using longitudinal data collected in 2019 and 2021 as part of the Household, Income and Labour Dynamics in Australia (HILDA) Survey, fixed-effects models of job satisfaction are estimated. Changes in the share of total weekly work hours usually worked from home are not found to have any significant association with changes in job satisfaction for men. By contrast, a strong significant positive (but nonlinear) association is found for women, and this relationship is concentrated on women with children. These findings suggest the main benefit of working from home for workers arises from the improved ability to combine work and family responsibilities, something that matters more to women given they continue to shoulder most of the responsibility for house and care work.

The COVID-19 pandemic and the associated social distancing policies saw a marked increase in the incidence of working from home in many countries during 2020 and 2021. In the United States, for example, the fraction of workers who report usually working from home in the previous week tripled between 2019 and 2021, rising from 5.7 to 17.9% (US Census Bureau 2022). It has been argued that this shift is the start of more lasting changes (e.g., Barrero, Bloom, and Davis 2021), with employers discovering potential productivity gains from moving to hybrid work arrangements that provide workers greater choice in where they work and employees attracted by potential lifestyle benefits.

Previous research has generally concluded that working primarily from home (also referred to as teleworking) is associated with higher levels of job satisfaction (Gajendran and Harrison 2007). Much of this research, however, has been conducted in an era when levels of teleworking were low. As already noted, persons who usually worked from home accounted for less than 6% of the US workforce in 2019. Similarly low levels also prevailed in other Western nations. In Australia, approximately 5% of workers worked mostly from home prior to the pandemic (Lim and Wooden 2020: 11). Likewise, the proportion of employed persons in 2019 recorded as “usually” working from home in the European Union (EU) averaged just 5.4% across EU member countries (Eurostat 2022).

The low prevalence of working from home pre-COVID suggests that previous research into the association between telework and job satisfaction may have been focused on selective sub-populations, raising the question of whether positive associations with job satisfaction will be found for affected populations that are much larger and that typically have not been provided with the opportunity to work remotely in the past. The opportunity to re-examine this question within such a novel setting has been provided by the COVID-19 pandemic.

In this study, we analyze the relationship between job satisfaction and working from home using data for Australia, a country where, in the wake of the pandemic, the incidence of working from home became far more widespread. Central to this study is the use of data from a panel survey that has for many years been collecting information from members of a large sample of Australian households about (among many other things) job satisfaction and usual hours of work and, most critically, how many of those hours are worked from home. We are thus able to examine how job satisfaction levels changed over time and the extent to which such change differed with the take-up, and extent of take-up, of home working. Critically, the study involves the examination of a large shock that resulted in a large increase in the extent of working from home over a short time window. This abrupt shift gives us good reason to be confident that our results are not confounded by other time-varying influences on job satisfaction.

## Theory and Hypotheses

### General Considerations

Following Locke’s value-percept discrepancy model (1969), job satisfaction results from a correspondence between what is valued in a job and what is actually received from that job. Building on this notion, we argue that the option to work from home is a job characteristic that is valued by many workers, and therefore providing workers with the option to work from home should result in higher job satisfaction.

The most obvious benefits for workers are the reduction in the monetary cost of, and time spent, commuting. The latter is particularly important given both the emotional strain associated with lengthy commutes (Golden 2006) and the time freed up for family and leisure pursuits (Gajendran and Harrison 2007; Laß and Wooden 2023). Working from home has also been associated with greater control over working schedules (Sardeshmukh, Sharma, and Golden 2012; Laß and Wooden 2023), making it easier to combine and balance work commitments with non-work activities. Relatedly, working from home may be associated with fewer meetings and interruptions (Wöhrmann and Ebner 2021), which may make for a less stressful work environment.

Working from home, however, has its downsides. It can be associated with a blurring of boundaries between work time and non-work time (Wöhrmann and Ebner 2021), making it more difficult to “turn work off” (Fan and Moen 2023) and facilitating work during so-called unsocial hours (i.e., evenings, nights, and weekends) (Laß and Wooden 2023). It also typically involves workers spending more time working on their own, which can lead to feelings of loneliness and isolation (Mann and Holdsworth 2003). Remote workers tend to receive less social support from co-workers and supervisors (Sardeshmukh et al. 2012; Wöhrmann and Ebner 2021). Related issues include “flexibility stigma” (e.g., Williams, Blair-Loy, and Berdahl 2013) and “proximity bias” (e.g., Williamson et al. 2022), with employees frequently reported to be hesitant to use telework for fear that career advancement prospects might be damaged (McCloskey and Igbaria 2003; Golden and Eddleston 2020).

Overall, working from home can be associated both with factors that enhance or diminish worker well-being. Nevertheless, if employees are given some freedom in deciding where to perform work, this should result in better matches between actual and desired working conditions and therefore increased job satisfaction.

Prior studies mostly support the assumption that working from home enhances job satisfaction, although the magnitude of association is often judged to be small. This includes both early small-scale studies, often based on non-representative samples (see the meta-analysis by Gajendran and Harrison 2007), as well as more recent studies based on large population-wide samples (e.g., Wheatley 2012, 2017; Dockery and Bawa 2014; Binder 2016; Kröll and Nüesch 2019; Reuschke 2019; Kim, Henly, Golden, and Lambert 2020; Bellmann and Hübler 2021; Yang, Kelly, Kubzansky, and Berkman 2023). That working from home is a valued job attribute is also suggested by recent surveys showing that many workers would like to continue to work from home after the pandemic (Barrero et al. 2021; Beck and Hensher 2021). We thus put forward the following hypothesis:

**H1:** Working from home will be positively associated with job satisfaction.

### The Moderating Roles of the Extent of Working from Home, Gender, Parenthood, and Lockdowns

The relationship between working from home and job satisfaction can be expected to vary with the extent of time spent working from home. Golden and Veiga (2005) argued that the loss of face time and the greater social isolation that accompany very high levels of telecommuting offset the benefits associated with working from home, and they hypothesized an inverted U-shaped relationship. Furthermore, at relatively low levels of remote working, additional hours worked from home may just reflect an increase in the amount of work done in the evenings or on weekends. In these situations, the many benefits from working from home may also be absent. Prior studies have shown that working from home that is driven by the need to catch up on work is indeed not associated with higher job satisfaction (Kim et al. 2020; Yang et al. 2023). Overall, the few studies that have investigated the relationship between the extent of working from home and job satisfaction confirm a nonlinear relationship (Golden and Veiga 2005; Virick, DaSilva, and Arrington 2010; Dockery and Bawa 2014). We therefore put forward the following hypothesis:

**H2:** The association between working from home and job satisfaction will resemble an inverse U-shape.

There may also be reasons to expect that the link between working from home and job satisfaction varies by gender. In most industrialized countries, women are the primary care givers and spend much more time on housework and childcare than do men (OECD 2021a). These different gender roles are reflected in the reasons women and men utilize working from home arrangements: Whereas men tend to work from home to accommodate job demands, women are more likely to work from home to accommodate family demands (Ammons and Markham 2004; Sullivan and Smithson 2007), and indeed spend significantly more time on housework and care when working from home (Powell and Craig 2015). Women have also been shown to benefit more from working from home in terms of reduced work–family conflict (Laß and Wooden 2023).

However, results from pre-COVID studies regarding gender differences in the association between working from home and job satisfaction are mixed. While some studies have found larger effects for women (e.g., Wheatley 2017; Reuschke 2019), others have found no gender differences (e.g., Wheatley 2012; Kim et al. 2020) or even that effects are larger or only present for men (Dockery and Bawa 2014; Binder 2016). We suspect that such mixed findings may be a function of the time of data collection, with working from home pre-pandemic often synonymous with long workweeks; hence, some of the benefits most valued by female workers (especially greater control over working schedules) were absent. Recent surveys, for example, have shown that women had a more positive experience with working from home during the pandemic than men had and were more likely to emphasize the benefit of having a more flexible work schedule (Beck and Hensher 2021; Ahmadi, Zandi, Cetrez, and Akhavan 2022). This outcome is despite (or maybe because) some studies show that women increased their unpaid labor time more than men did during the pandemic (e.g., Craig and Churchill 2021; Yaish, Mandel, and Kristal 2021), and this was particularly the case when working from home (Yaish et al. 2021). Despite this extra workload, many women may still have seen the flexibility associated with working from home in a positive light: At the very least, it allowed them to accommodate the increased housework burden more easily than when working on-site. Similarly, working from home enabled women to react to unforeseen childcare demands arising from daycare or school closures, or when children need to be isolated after testing positive for COVID-19. By contrast, men were more likely to voice excitement about returning to the office after COVID restrictions eased, missing in-person collaboration with colleagues and a work environment free of distractions (Mattey et al. 2020). Overall, we expect the benefits of working from home for women in terms of fulfilling family demands to outweigh any benefits for men, leading to the following hypothesis:

**H3:** The positive association between working from home and job satisfaction will be stronger for women.

For similar reasons, we also expect differences between parents and childless workers. Since parents have more extensive family demands, they should particularly benefit from the improved ability to balance work and family commitments that comes with working from home, increasing overall job satisfaction. In line with this assumption, parents have been reported to be more likely to emphasize spending more time with family as an advantage of working from home during COVID, although they were also more likely to report interruptions from family as a challenge (Beck and Hensher 2021). While both mothers and fathers should benefit from working from home to accommodate family demands, the benefits may be more pronounced for mothers given their higher unpaid workload and thus greater dependence on flexible working conditions.

**H4a:** The positive association between working from home and job satisfaction will be stronger for mothers than for childless women.
**H4b:** The positive association between working from home and job satisfaction will be stronger for fathers than for childless men.

Finally, we expect the benefits of working from home to vary between workers living in regions where lockdowns were in place and those living elsewhere. Outside lockdown areas, working from home was an option available to workers (and to many more than was the case pre-pandemic), whereas during lockdowns, workers whose jobs could be done from home were forced to do so. We assume the match between desired and actual job characteristics to be better for workers who deliberately chose to work from home. For example, enforced working from home may result in workers working from home who are strongly dependent on interactions with co-workers, experience frequent interruptions from others at home, or do not have an adequate workspace at home.

Not only the freedom of workplace choice but also the context in which workers performed their work from home differed between lockdown and non-lockdown areas. For example, during lockdown, personal contacts were restricted, potentially leading to increased feelings of isolation if working from home, particularly among those living alone. Furthermore, access to formal childcare and schools was greatly restricted during lockdown. Many parents thus had to undertake work while simultaneously attending to the needs of their children for extended periods. These circumstances bring us to our final hypothesis:

**H5:** The positive association between working from home and job satisfaction will be stronger for persons residing in areas without prolonged lockdowns.

## The Australian Context

**Figure 1.**
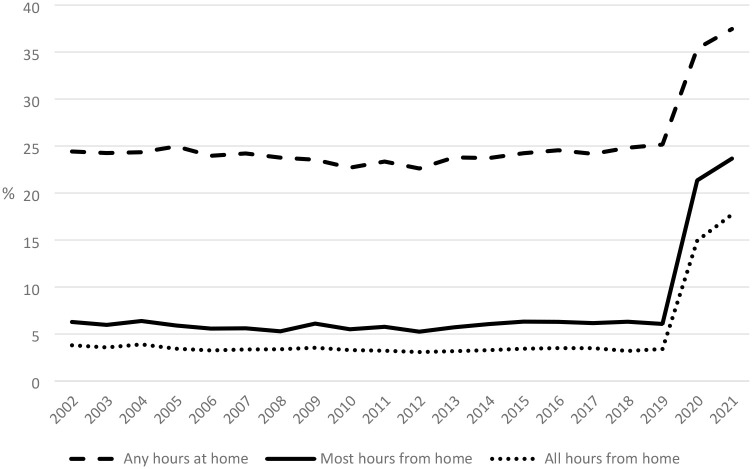
Extent of Working from Home (% of Employed Persons), Australia, 2002–2021 *Source:* HILDA Survey release 21 (Department of Social Services / Melbourne Institute of Applied Economic and Social Research 2022). *Notes:* Estimates are population weighted.

According to the Household, Income and Labour Dynamics in Australia (HILDA) Survey, approximately one in four Australian workers in 2019 usually worked at least some hours at home, and this level had changed very little over the preceding two decades (see Figure 1). Much of this working from home activity, however, appears to have been of the “take work home with you” variety,^1^ with, as noted earlier, a much smaller fraction of the workforce—approximately 6%—estimated to work most of their usual work hours from home, and only about half of this group worked all hours from home. Furthermore, the majority of those working mostly from home—slightly more than 3 in 4—were self-employed. The proportion of employees who worked primarily from home was typically no more than 1.5%. This despite Australia being one of the few OECD countries where legislation provides certain groups of employees with the right to request flexible workplace arrangements, including work location (OECD 2021b: 297).

With the advent of the COVID-19 pandemic, however, the incidence of working from home in Australia rose sharply. As shown in Figure 1, the proportion of employed persons working at least 50% of their usual weekly paid hours at home rose from just 6.1% in 2019 to 21.3% in 2020 and to 23.7% in 2021. A key driver of this change was the government response to containing the spread of the virus, which from the outset in late March 2020 included advice to work from home wherever possible (for an overview of initial responses by Australian Governments to the pandemic, see Stobart and Duckett 2022). This approach was a key element in the desire by governments to reduce population movement, which in turn was central to Australia’s zero-COVID strategy. Note there were also periods when some state governments, in response to outbreaks of the virus, imposed stringent lockdown measures. During these lockdowns, people were permitted to leave home for only four reasons: 1) shopping for essentials; 2) outdoor exercise and recreation (and then only within a 5 km radius of their home); 3) attending medical appointments or providing care to others; or 4) working or studying if not feasible from home. Most children were required to learn at home, with only children of essential workers permitted to physically attend school. Similar restrictions applied to childcare centers, assuming they opened at all; the risk of COVID-19 transmission saw many close for extended periods.

Victoria, Australia’s second most populous state, was most affected, with its citizens subject to seven periods of lockdown. They experienced two prolonged lockdowns, which for residents of Melbourne, the state’s largest city, commenced in July 2020 and lasted almost four months, and then again about a year later in August 2021, lasting 2.5 months. Similarly, residents of some parts of Australia’s largest city, Sydney, were subject to progressive lockdowns from late June 2021, before a citywide lockdown was imposed in early August and not lifted until mid-October. Shorter lockdowns were also imposed on residents of New South Wales and Victoria who lived outside of the two major cities.

As in other countries, the policy responses to the pandemic, and especially the closure of many businesses, saw a marked rise in the unemployment rate from a seasonally adjusted 5.1% in February 2020 to 7.0% six months later (ABS 2023a). This rise, however, was temporary. By August 2021, when wave 21 of the HILDA Survey went into the field, the unemployment rate had fallen below pre-pandemic levels (4.6%), and over the year ahead would fall further. Indeed, the combination of expansive fiscal and monetary policies with international border restrictions led to a labor market that by the second half of 2021 (and despite the continued lockdowns) was characterized by labor shortages. This trend was reflected in the number of job vacancies, which rose by 78% between February 2020 and November 2021 (ABS 2023b). Such developments might be expected to result in greater pressures being placed on employees to work both longer hours and more intensely. The evidence, however, does not support this hypothesis, with HILDA Survey data showing a significant decline in self-reported work pace and intensity between 2019 and 2021.

Also relevant for this analysis is the gender division of paid and unpaid work. While gender role attitudes have become more egalitarian in Australia over the past two decades (Wilkins, Vera-Toscano, Botha, and Dahmann 2021), the actual division of labor is still highly gendered, particularly among parents. The maternal employment rate (69%) is below the OECD average (71%), and more than half of mothers work part-time (OECD 2023a: chart LMF1.2.A). By contrast, women and especially mothers do a much larger share of housework and care than do men (Wilkins and Laß 2018; OECD 2021a). As previously mentioned, it was also women who shouldered most of the extra unpaid work during lockdowns, although men also invested more time in childcare (Craig and Churchill 2021). Among the factors reinforcing traditional gender roles in Australia are high costs of formal childcare. For example, childcare costs for a two-parent household in which both parents earn the average wage amounted to 22% of net household income in 2022, considerably higher than the OECD average of 13% (OECD 2023b).

## Data and Methods

### Survey Data

The data we use are from the HILDA Survey, a longitudinal study following members of a nationally representative sample of Australian households on an annual basis since 2001 (Watson and Wooden 2021). Response rates are relatively high, especially the annual re-interview rate, which rose from 87% in wave 2 to more than 94% by wave 5 and has remained above that level in every wave since (Summerfield et al. 2022: 172). Thus, whereas non-response means the sample will not precisely match the wider Australian population, differences should be relatively small. There is, however, one weakness in the design of the HILDA Survey: Without constant refreshment samples (and one was added in wave 11), the study cannot adequately represent migrants entering Australia after the panel commenced. Immigrants are thus under-represented: Just 18% of employed respondents in the HILDA Survey sample in wave 21 were born overseas, far less than the 32% recorded in the 2021 Population Census. Despite this, on most other characteristics (such as age, occupation, and industry) the distribution of HILDA Survey respondents aligns closely with the distribution in the population.^2^

The sample used for this analysis is persons observed in paid employment at time of interview in both waves 19 (conducted mostly in 2019 and thus prior to the COVID-19 pandemic) and 21 (conducted mostly in the latter half of 2021 and thus coinciding with lockdowns in New South Wales and Victoria). Full-time students were excluded. This sample provided a total of 15,756 observations from 7,878 unique individuals. Missing observations on covariates, however, reduced the size of the sample available for analysis to a maximum of 14,734 observations (from 7,367 individuals).

### Variables

#### Dependent Variable

The outcome variable is a self-reported measure of overall job satisfaction scored on a 0 to 10 scale where the end points are labeled “totally dissatisfied” and “totally satisfied.” The specific question asked is: “All things considered, how satisfied are you with your job?”

#### Key Independent Variable: Working from Home

Each year, survey respondents that reported either doing any work in a job, business, or farm during the previous seven days, or being away from a job, business, or farm (e.g., because of holidays or sickness), are asked how many hours they usually worked each week in their main job, and of those how many hours are usually worked at their home. From these responses, we calculated the proportion of usual weekly working hours that are worked at home.

The focus of these questions on usual hours of work, however, is potentially problematic, with some respondents possibly interpreting “usual” as referring to life prior to the pandemic. This concern was partly addressed by the inclusion of an interviewer note explaining that “usual” referred to a respondent’s current working situation and not their working situation pre-pandemic. This note, however, only helps if the respondent queries what is meant by the term “usual.” The data collected during the pandemic, but especially in 2020, may thus understate both the number of persons working any hours from home and the number of hours per week that were worked from home. By 2021, however, this risk of understatement will have diminished considerably, given workers will have become used to their pandemic working patterns.

#### Control Variables

Selection of control variables was guided by previous analyses of job satisfaction using HILDA Survey data, and especially Green, Kler, and Leeves (2010), Dockery and Bawa (2014), and Buddelmeyer, McVicar, and Wooden (2015). We thus include controls for age group (six categories), marital/partnership status (three categories), the presence of children differentiated by the age and economic dependency of the youngest child (five categories),^3^ educational attainment (four categories), the presence of a restrictive long-term health condition or disability, employment status/contract type (five categories), length of tenure with the current employer (six categories), occupation (eight categories), hours usually worked per week (specified as a quadratic), whether a multiple jobholder, supervisory responsibilities, membership of a trade union, public-sector employment, employer size (i.e., number of employees) (five categories), industry (19 categories), region of residence (i.e., remoteness area,^4^ three categories), state or territory (eight categories), survey wave, presence of another adult during the interview, and whether the interview was conducted in person or by telephone. Potential gender differences are accounted for by estimating separate models for men and women.

A list of variables included in the analysis, along with their unweighted means, is presented in Table 1. The table shows that between 2019 and 2021, average job satisfaction levels rose by a modest 0.15 and 0.12 points on the 11-point scale for men and women, respectively. Simultaneously, the percentage of workers who worked most of their hours from home increased considerably, from 5 to 21% for men and from 8 to 28% for women.^5^

**Table 1.** List of Variables and (Unweighted) Mean Values

Variable	Women		Men	
	2019	2021	2019	2021
**Outcome variable**				
Overall job satisfaction (0–10)	7.76	7.88	7.78	7.93
**Working from home**				
Hours usually worked from home per week (no.)	3.319	10.270	3.322	9.295
Any hours worked from home	0.283	0.437	0.277	0.403
50% or more of total hours worked from home	0.080	0.284	0.054	0.215
Proportion of hours worked from home	0.107	0.296	0.080	0.232
Share of total hours usually worked from home (%)				
None	0.717	0.563	0.723	0.597
1–19	0.112	0.065	0.148	0.113
20–39	0.077	0.067	0.065	0.055
40–59	0.026	0.042	0.021	0.035
60–79	0.011	0.028	0.009	0.024
80–99	0.009	0.024	0.008	0.018
100	0.048	0.211	0.027	0.158
**Age group (years)**				
15–24	0.082	0.046	0.077	0.043
25–34	0.254	0.237	0.271	0.243
35–44	0.227	0.230	0.227	0.245
45–54	0.229	0.234	0.216	0.220
55–64	0.174	0.197	0.169	0.191
65 or older	0.035	0.056	0.041	0.059
**Marital / partnership status**				
Single	0.283	0.271	0.237	0.224
Married	0.514	0.529	0.550	0.569
Cohabiting	0.204	0.200	0.213	0.207
**Age of youngest child (interacted with dependence)**				
Aged 0–4 years	0.148	0.146	0.185	0.189
Aged 5–14 years	0.210	0.215	0.192	0.192
Dependent child aged 15–24 years	0.084	0.083	0.064	0.068
Independent child	0.068	0.071	0.050	0.059
No children	0.489	0.485	0.509	0.493
**Health status**				
Restrictive long-term health condition or disability	0.096	0.109	0.077	0.090
**Educational attainment**				
Year 11 and below	0.107	0.104	0.133	0.130
Year 12	0.131	0.122	0.145	0.139
Vocational qualification or diploma	0.320	0.327	0.411	0.416
Bachelor’s degree or higher	0.442	0.447	0.311	0.315
**Employment type**				
Permanent employee	0.648	0.700	0.642	0.664
Fixed-term contract employee	0.113	0.081	0.074	0.052
Casual employee	0.130	0.105	0.103	0.082
Self-employed	0.105	0.111	0.179	0.201
Other	0.004	0.003	0.002	0.002
**Tenure with current employer (years)**				
Less than 1	0.168	0.147	0.149	0.130
1 to <2	0.086	0.046	0.085	0.057
2 to <5	0.255	0.256	0.244	0.244
5 to <10	0.207	0.233	0.207	0.220
10 to <20	0.192	0.200	0.196	0.213
20 or more	0.092	0.118	0.120	0.137
**Occupation**				
Managers	0.123	0.126	0.195	0.201
Professionals	0.345	0.353	0.225	0.227
Technicians & trades workers	0.042	0.041	0.212	0.210
Community & personal service workers	0.147	0.145	0.066	0.062
Clerical & administrative workers	0.206	0.205	0.061	0.060
Sales workers	0.076	0.071	0.040	0.041
Machinery operators & drivers	0.011	0.013	0.109	0.112
Labourers	0.050	0.047	0.091	0.087
**Other job characteristics**				
Usual hours worked per week in all jobs	32.97	33.11	42.08	41.25
Multiple job holder	0.088	0.083	0.067	0.065
Normally supervise work of other employees	0.417	0.417	0.527	0.506
Trade union member	0.218	0.223	0.163	0.159
Public sector	0.315	0.322	0.180	0.173
**Firm size (# of employees)**				
Small (0–19)	0.230	0.237	0.315	0.326
Medium (20–99)	0.122	0.120	0.140	0.139
Large (100–499)	0.116	0.116	0.116	0.122
Very large (500 or more)	0.487	0.467	0.403	0.376
Firm size unknown	0.045	0.061	0.026	0.036
**Industry**				
Agriculture, forestry & fishing	0.015	0.015	0.038	0.040
Mining	0.006	0.007	0.035	0.034
Manufacturing	0.039	0.034	0.114	0.109
Electricity, gas, water & waste services	0.006	0.007	0.019	0.018
Construction	0.016	0.018	0.148	0.154
Wholesale trade	0.019	0.019	0.042	0.044
Retail trade	0.090	0.083	0.061	0.058
Accommodation & food services	0.045	0.037	0.030	0.025
Transport, postal & warehousing	0.022	0.021	0.067	0.064
Information media & telecommunications	0.012	0.012	0.013	0.014
Financial & insurance services	0.040	0.041	0.033	0.036
Rental, hiring & real estate services	0.015	0.017	0.013	0.012
Professional, scientific & technical services	0.082	0.076	0.089	0.097
Administrative & support services	0.031	0.028	0.028	0.030
Public administration & safety	0.072	0.078	0.079	0.075
Education & training	0.161	0.163	0.059	0.057
Health care & social assistance	0.284	0.301	0.074	0.077
Arts & recreation services	0.013	0.015	0.018	0.018
Other services	0.031	0.031	0.039	0.039
**Geographical location**				
Major city	0.636	0.628	0.632	0.620
Inner regional	0.255	0.262	0.256	0.267
Outer regional or remote	0.109	0.110	0.112	0.113
**State**				
New South Wales	0.278	0.280	0.283	0.283
Victoria	0.261	0.257	0.260	0.259
Queensland	0.217	0.217	0.220	0.221
South Australia	0.086	0.086	0.082	0.084
Western Australia	0.092	0.093	0.090	0.091
Tasmania	0.035	0.036	0.034	0.035
Northern Territory	0.008	0.007	0.008	0.006
Australian Capital Territory	0.024	0.024	0.023	0.022
**Interview characteristics**				
Other adults present during the interview	0.272	0.177	0.332	0.208
Interviewed by phone	0.093	0.780	0.098	0.770
Observations	3,570	3,570	3,797	3,797

### Analytical Approach

We begin with a simple model in which job satisfaction (*JS*) is a function of the amount (or share) of working time worked from home (*WFH*) and a set of other observable individual-level characteristics (*X*). This model takes the form:

(1) $$JS_{it}=\alpha_{0}+\alpha_{1}{WFH}_{it}+\alpha_{2}X_{it}+\varepsilon_{it}$$

We are interested in the change in job satisfaction (Δ*JS_i_*), and so specify a first-differences model:

(2) $${\Delta JS}_{i}=\beta_{0}+\beta_{1}\Delta {WFH}_{i}+\beta_{2}\Delta X_{i}+\Delta \varepsilon_{i}$$

When the number of time periods equals 2, this is identical to a fixed-effects model:

(3) $${JS}_{it}=\delta_{0}+\beta_{1}{WFH}_{it}+\beta_{2}X_{it}+\varepsilon_{it}+\mu_{i}$$

We experiment with various functional forms for *WFH*, including dummy variables identifying whether any or most hours are worked from home, a continuous variable measuring the proportion of total work hours that are worked from home, and a categorical variable resulting from splitting the continuous variable into seven levels of working from home.

Estimation is undertaken using the xtreg command in Stata (version 16).

## Results

### Main Models

Table 2 presents results from linear fixed-effects models in which we regressed job satisfaction on several measures of working from home separately by gender. All models accounted for the control variables listed earlier. For reasons of brevity, however, only the coefficients of interest are reported here (estimates from the full models are reported in Tables A.4 and A.5). Starting with the simple binary measure of whether workers do any of their usual hours at home (model (1)), there was a significant positive association with job satisfaction for women. The coefficient of 0.220 means that female workers who moved from working no hours at home in 2019 to working some hours at home in 2021 experienced, on average, more than a fifth of a point increase on the 0 to 10 job satisfaction scale. By contrast, the coefficient for men, while also positively signed, was much smaller (0.069) and statistically insignificant. A very similar pattern arose when we considered the effect of working 50% or more of the usual working hours from home (model (2)) or a linear specification of the proportion of paid hours worked from home (model (3)). H1 can therefore be supported only for women.

**Table 2.** Working from Home and Overall Job Satisfaction (Fixed-Effects Regression Results)

Model no.	Working from home variable	Women			Men			Significance of interaction: WFH & gender
		Coefficient (robust SE)	R-squared	Rho	Coefficient (robust SE)	R-squared	Rho	
(1)	Any hours worked from home	0.220*** (0.060)	0.056	0.616	0.069 (0.053)	0.053	0.611	*
(2)	50% or more of hours worked from home	0.247*** (0.064)	0.056	0.616	0.059 (0.063)	0.053	0.610	*
(3)	Proportion of hours worked from home	0.236** (0.073)	0.055	0.615	0.037 (0.074)	0.052	0.610	*
(4)	Share of hours worked from home (%) (ref. = 0)							
	1–19	0.129 (0.080)	0.059	0.616	0.111 (0.064)	0.054	0.611	n.s.
	20–39	0.201* (0.094)			−0.085 (0.088)			*
	40–59	0.314* (0.137)			0.170 (0.112)			n.s.
	60–79	0.607*** (0.178)			0.213 (0.128)			n.s.
	80–99	0.382* (0.156)			0.085 (0.184)			n.s.
	100	0.202** (0.076)			0.043 (0.079)			n.s.
(5)	Proportion of hours worked from home	1.365*** (0.355)	0.058	0.616	0.043 (0.317)	0.052	0.610	**
	Proportion of hours worked from home squared	−1.137** (0.348)			−0.005 (0.310)			*
Observations		7,140			7,594			

*Notes:* All models include controls as described in text and listed in Table 1. Complete results are reported in Tables A.4 and A.5. n.s., not significant; WFH, working from home.

* *p* <0.05; **p <0.01; ***p <0.001.

We next tested for nonlinearity in the association between the extent of working from home and job satisfaction. First, we distinguished between different shares of time worked from home using seven categories (model (4)). For women, we found significant associations between almost every share of time worked from home and job satisfaction. However, as expected (H2), the magnitudes of the coefficients implied a nonlinear relationship, with job satisfaction increasing across categories until the 60 to 79% category (which was associated with a 0.607-point increase) before declining as the working from home share rose further (only amounting to a 0.202-point increase for those working exclusively from home). Second, we used a more parsimonious quadratic specification (model (5)). The results from the estimation of this alternative specification confirmed the nonlinear shape of the relationship for women, with the linear term positive and significant and the quadratic term negative and equally significant. Again, we found no significant associations for men in either model.

These findings already provide support for our assumption that women benefit more than men from working from home in terms of job satisfaction (H3). However, we also formally tested for significant gender differences by re-running our analyses on the pooled model of men and women and including interaction terms between the working from home measures and being female. In all but one model, the interaction terms were significant, suggesting that working from home is indeed associated with significantly larger gains in job satisfaction for women than for men. The exception is model (4), in which only one of the interaction terms was significant (though two more were at the 10% significance level). This lack of significance may at least in part be attributed to the loss of statistical power arising from splitting working from home into seven categories.

We suspected that the greater ability to combine work with family demands was driving the positive association between working from home and job satisfaction, and that the association should therefore be stronger for mothers than for non-mothers (H4a) and for fathers than for non-fathers (H4b). To corroborate these assumptions, we re-ran our detailed categorical model (model (4) in Table 2) separately for individuals living with and without own children in the household. Results are presented in Table 3 (with full models in Table A.6). For women, we found that several of the coefficients were considerably larger for mothers than for women without children. In particular, working between 60 and 79% from home was associated with a significant increase in job satisfaction of nearly one point (0.940) for mothers, whereas among childless women the coefficient was only 0.231 and statistically insignificant. Further analyses using a joint model for all women and including an interaction term for having children showed that the coefficients for the categories 1 to 19% and 60 to 79% were significantly larger among mothers than non-mothers. A similar but much weaker pattern arose for men: Working 1 to 19% or 60 to 79% of the time from home was significantly and positively associated with job satisfaction among fathers, whereas working from home was not associated with job satisfaction among childless men. Yet, the coefficients were mostly much smaller than for mothers. Furthermore, analyses based on interaction models showed that none of the working from home coefficients for men differed significantly between fathers and childless men. Overall, our findings thus supported H4a but provided only very limited support for H4b.

**Table 3.** Impact of Children on the Relationship between Working from Home and Overall Job Satisfaction (Fixed-Effects Regression Results)

Share of hours worked from home (%) (reference group = 0)	Women			Men		
	Without children	With children	Significance of interaction: WFH & having children	Without children	With children	Significance of interaction: WFH & having children
1–19	0.016 (0.128)	0.220* (0.110)	*	−0.043 (0.108)	0.234** (0.084)	n.s.
20–39	0.143 (0.165)	0.210 (0.130)	n.s.	−0.251 (0.159)	−0.053 (0.111)	n.s.
40–59	0.207 (0.194)	0.230 (0.192)	n.s.	0.069 (0.190)	0.247 (0.147)	n.s.
60–79	0.231 (0.270)	0.940*** (0.246)	*	0.137 (0.255)	0.342* (0.166)	n.s.
80–99	0.265 (0.289)	0.569** (0.216)	n.s.	−0.010 (0.331)	0.225 (0.265)	n.s.
100	0.235 (0.126)	0.160 (0.107)	n.s.	0.004 (0.115)	0.109 (0.119)	n.s.
Joint significance (*p* values)	0.551	0.003		0.749	0.036	
*R*-squared	0.062	0.104		0.083	0.090	
Rho	0.619	0.745		0.652	0.691	
Observations	3,480	3,660		3,802	3,792	

*Notes:* Robust standard errors in parentheses. Control variables are the same as in Table 2. Complete results are reported in Table A.6. n.s., not significant; WFH, working from home.

* *p* <0.05; **p <0.01; ***p <0.001.

Finally, we tested whether the strength of the associations varied between states that were in prolonged lockdowns in 2021 and those that were not. For reasons of space, and given that our main analyses had revealed significant associations only for women, we report only results of these additional analyses for this sub-group. Results are reported in models (1) and (2) of Table 4 (full models in Table A.7).

**Table 4.** Further Analyses and Robustness Checks: Working from Home and Overall Job Satisfaction, Women

Share of hours worked from home (%) (reference group = 0)	Lockdown states (NSW + VIC) (1)	Other states (2)	Significance of interaction: WFH & lockdown states	WFH-intensive occupations (3)	Other occupations (4)	Significance of interaction: WFH & WFH-intensive occupations	FE ordered logit (5)
1–19	0.186 (0.098)	0.031 (0.133)	n.s.	0.250 (0.143)	0.009 (0.097)	n.s.	0.252 (0.157)
20–39	0.331** (0.113)	0.012 (0.161)	n.s.	0.327* (0.156)	0.167 (0.124)	n.s.	0.354* (0.175)
40–59	0.198 (0.163)	0.464* (0.226)	n.s.	0.265 (0.193)	0.216 (0.195)	n.s.	0.550** (0.209)
60–79	0.726** (0.228)	0.485 (0.280)	n.s.	0.917*** (0.244)	0.395 (0.274)	n.s.	1.114*** (0.305)
80–99	0.302 (0.195)	0.661* (0.260)	n.s.	0.552* (0.224)	−0.032 (0.225)	*	0.792** (0.298)
100	0.168 (0.086)	0.387* (0.191)	n.s.	0.061 (0.140)	0.274** (0.105)	n.s.	0.381** (0.144)
Joint significance (*p* values)	0.008	0.024		<0.001	0.093		<0.001
*R*-squared	0.094	0.079		0.174	0.064		
Rho	0.647	0.685		0.784	0.653		
Observations	3,839	3,301		1,809	5,295		4,574

*Notes:* Models (1) to (4) report coefficients (and robust standard errors in parentheses) from linear fixed effects regression models. Model (5) reports coefficients from a fixed-effects ordered logit regression. Control variables are the same as in Table 2. Complete results are reported in Tables A.7 and A.8. n.s., not significant; WFH, working from home.

* *p* <0.05; **p <0.01; ***p <0.001.

We found working from home to be associated with increased job satisfaction in both lockdown states and other states, but the two sets of coefficients were far from identical. In lockdown states, working 100% from home was associated with a smaller (and insignificant) increase in job satisfaction than in the other states. This outcome might be the result of many workers in these states being forced to work entirely from home. By contrast, the relatively few workers in lockdown states who reported working 60 to 79% of total working hours from home experienced a larger increase in job satisfaction than workers working a similar pattern in non-lockdown states. When focusing only on states that were not subject to lockdowns in 2021, and hence where working from home was far less likely to be the result of a directive from government, we found two distinct groups separated at the 40% of work time worked at home cut-off (i.e., two days per week for those working a standard five-day week). Women who worked at home less often than this had job satisfaction levels in 2021 that were no different from when they did not work any hours from home in 2019. In contrast, for women whose working from home share exceeded this cutoff, job satisfaction was enhanced by about half a point, and the proportion of hours worked at home beyond this threshold mattered little. Our finding that women’s job satisfaction was enhanced by working from home remains intact, but how that relationship varies with the proportion of time worked from home may differ somewhat from what was initially suggested. Despite these dissimilar patterns, an additional interaction model showed that none of the working from home coefficients differed significantly between lockdown states and other states, so we cannot confirm H5 with confidence.

### Robustness Checks

We estimated three additional modified versions of our detailed categorical model with the aim of testing the robustness of our results, focusing again on women. First, we tested whether our results changed if we focused on those occupations for which working from home is most feasible. We thus re-estimated our models using the subsample of persons employed in occupations for which the intensity of working from home is high (“WFH intensive”). To identify these WFH-intensive occupations, we used data on method of travel to work from the 2021 Census of Population (ABS 2022), which coincided with fieldwork for wave 21 of the HILDA Survey. This information provided estimates of the proportion of employed persons within each of 358 occupation unit groups who worked at home on the day of the Census that could then be matched to the occupation data collected in the HILDA Survey. Estimates ranged from zero (e.g., forklift drivers, waiters, kitchen hands) to 87% (authors and book and script editors). We then defined a WFH-intensive occupation as one in which at least 40% of employed persons in that occupation worked from home on Census day. This threshold was exceeded in 82 occupation groups, representing 23.4% of Australian workers.

Focusing on this subgroup of occupations yielded much stronger associations between working from home and job satisfaction for women (see model (3) in Table 4). For example, a working from home share of 60 to 79% was associated with a 0.917-point increase in this subgroup, which compares to the 0.607 increase for all women reported in Table 2. Somewhat unexpectedly, working exclusively from home was not associated with any significant improvement in job satisfaction. We speculate that this might be the result of many women in this group being forced to work from home (because of lockdowns in New South Wales and Victoria). Few significant associations were found for men (see Table A.8).

Second, since the outcome variable involves discrete values bounded between 0 and 10, it could be argued that an estimator designed for ordinal dependent variables is more appropriate than linear regression. We thus re-estimated our preferred model using a fixed-effects estimator developed by Baetschmann, Ballantyne, Staub, and Winkelmann (2020) for the conditional ordered logit case (feologit). The results are reported in model (5) in Table 4. While the coefficients from the fixed-effects linear model (Table 2) are not directly comparable with those of the fixed-effects ordered logit model, since they are scaled differently, a comparison of the pattern is valid. Just like the linear case, the ordered logit results revealed a positive association between the share of time worked from home and job satisfaction for women that increased until the 60 to 79% category and then declined. Additionally, we can compare ratios of coefficients, since the ratio of two coefficients is the estimate for the ratio of two average marginal effects (AME) for both the fixed-effects linear model and the fixed-effects ordered logit model. For example, comparing the coefficient on “40 to 59%” to the one on “60 to 79%” gives a ratio of 0.52 (0.314/0.607) for the linear model (in Table 2) and 0.49 (0.550/1.114) for the ordered logit model: In both regressions, the AME of the variable “60 to 79%” is approximately twice as large as the AME of the variable “40 to 59%.” In short, use of an estimator designed for an ordinal outcome variable makes no difference to our conclusions.

Finally, we accounted for the possibility that the number of working hours may serve as a mediator linking working from home and job satisfaction. As can be seen from Tables A.2 and A.3, working a small extent from home was associated with considerably longer working hours than both never working from home and working from home to a great extent. These differences may, in turn, affect job satisfaction. Therefore, it could be argued that the number of working hours should not be controlled for in the models. Re-running the categorical model after dropping the number of working hours and its square from the list of control variables, however, produced very similar results for both men and women.

## Discussion

One consequence of the COVID-19 pandemic has been the marked rise in the incidence of working from home and the possibility that this transformation in the way many people work is permanent (Barrero et al. 2021). One reason why the incidence of working from home is not expected to revert to pre-pandemic levels is that working from home provides benefits to workers that those workers will be reluctant to forego. The analysis reported in this article suggests this hypothesis is only true for women. For men, we could not find any evidence that the marked growth in working from home between 2019 and 2021 in Australia has been associated with a change in job satisfaction levels on average. By contrast, the magnitude of the estimated effects of working from home on women’s, and particularly mothers’, job satisfaction was not small. Coefficients in the order of 0.9 (obtained for mothers spending 60 to 79% of paid hours at home) are relatively large when judged against an outcome variable with a standard deviation of approximately 1.5.

The gender difference uncovered in this research is something that sets our study apart from previous research (e.g., Gajendran and Harrison 2007; Dockery and Bawa 2014). This discrepancy may reflect at least two trends specific to the pandemic period. First, there are marked differences in the types of jobs for which opportunities to work from home were possible. As noted earlier, prior to the pandemic, the large majority of Australians who worked primarily from home were self-employed. Very few employees (less than 1.5%) were given the opportunity to work primarily from home, and working from home was associated with long workweeks.

Second, because of reduced childcare availability and the shift to remote learning, the demand for unpaid work increased substantially in the COVID-19 period. In this context, working from home could be a means for parents to accommodate this increased workload, but one that was not equally important for both genders: Women usually assume a much larger share of unpaid work than men, and they also did more of the additional care work during the pandemic (Craig and Churchill 2021). Thus, working arrangements that better allow for the caring role, such as working from home, should have mattered far more to women, and especially to mothers. By contrast, for men, who are more likely to utilize working from home in the interest of work performance, the perks from this working arrangement (e.g., reduced commuting time) must have been offset by the disadvantages (e.g., working in isolation or diminished promotion prospects).

These gender differences also suggest that working from home could be yet another factor exacerbating the gender divide in the labor market. If working from home becomes a much more common working arrangement in the post-pandemic era, as is often argued, our results suggest that women are more likely than men to take advantage of it. On one hand, working from home could allow many women with care responsibilities to be employed in the first place or to extend their working hours. On the other hand, working from home could be a factor that will further widen the gender wage gap. This widening gap might occur if wages adjust to compensate for the non-wage benefits of working from home. Workers have reported they are prepared to forfeit a significant fraction of their pay in return for the ability to work from home (e.g., Mas and Pallais 2017; Barrero et al. 2021). But perhaps more crucially, in organizations where workers have the flexibility to choose where to work, people who choose to work more often from home may, because of flexibility stigma, be more likely to be overlooked for pay rises, promotions, and other opportunities that enhance career progression (Golden and Eddleston 2020).

The estimates obtained also suggest that the relationship between the amount of time worked from home and job satisfaction is nonlinear. In particular, many of the specifications imply that job satisfaction is greatest for women who work the majority of their time from home, but not all of their time. This outcome suggests that the optimal working arrangement, at least from the employee’s perspective, is a hybrid one involving some days spent in the traditional workplace. Nevertheless, this finding was not obvious once we limited our analysis to workers living in states without lockdowns. It thus may be that the disadvantages associated with working entirely at home are only pronounced when workers are required to spend not only all their working time at home but also most of their social life. By contrast, in non-lockdown states where people were less restrained regarding both place of work and leisure activities, job satisfaction appears to plateau after a certain threshold of working from home is reached, a finding that resembles that of Golden and Veiga (2005).

We also recognize that while the data we use have a number of strengths (notably they provide observations from the same workers collected in 2019, prior to the pandemic, and in 2021, and are drawn from a national probability sample), they are not without limitations. First, survey data are self-reported and thus subject to measurement error. In particular, we were concerned about the possibility that hours worked at home were being systematically understated, especially by workers living under lockdown restrictions at the time of being interviewed. That said, restricting the sample to those residing in non-lockdown states did not produce results that were vastly different. Second, the data provide information on only the number of hours worked at home in a usual week; we cannot distinguish part days worked at home from full days. Third, while we describe the underlying sample as nationally representative, the longitudinal nature of the sample design means recent immigrants are underrepresented.

## Conclusion

Using household panel survey data from a sample originally selected to be representative of the Australian population and consistent with data from other sources both in Australia and in other countries, we reported evidence of a marked rise in the proportion of time worked from home during the COVID-19 pandemic. More important, we found this growth in the prevalence of working from home associated with a large rise in reported job satisfaction among women. Among men there was no such association. We also found that this rise in overall job satisfaction among women was most marked for those with children and was thus likely a function of the higher flexibility available to balance work and non-work commitments. The relationship between working from home and job satisfaction does not, however, appear to be linear, though the precise nature of the relationship is still unclear. Among female workers residing in states affected by government-mandated lockdowns at the time of data collection in 2021, effects were largest for women spending between 60 and 79% of their usual paid hours working at home. In contrast, for those workers residing in states not directly impacted by lockdowns, the job satisfaction benefits were much the same for all women working 40% or more of their hours from home.

Overall, this study shows that the new way of working brought on by the COVID-19 pandemic can benefit worker well-being, but more so for certain groups, most notably women with children. It is up to future research to establish whether these associations will persist. It is possible, for example, that for many workers, being newly exposed to working from home may have led to an upward bump in reported job satisfaction that was only temporary. If, however, the beneficial effects for job satisfaction do persist, more women (and particularly mothers) can be expected to sort into home working arrangements, potentially enhancing existing gender inequalities in both employment careers and household responsibilities. Avoiding these downside risks will likely require further changes to the way we work, to workplace culture, and to gender norms around paid and unpaid work.

## Supplemental Material

sj-pdf-1-ilr-10.1177_00197939241301704 – Supplemental material for Working from Home, COVID-19, and Job Satisfaction Supplemental material, sj-pdf-1-ilr-10.1177_00197939241301704 for Working from Home, COVID-19, and Job Satisfaction by Inga Laß, Esperanza Vera-Toscano and Mark Wooden in ILR Review

## Footnotes

1 Survey data collected by the Australian Bureau of Statistics (ABS 2021a) prior to the pandemic reveal that between 38% and 43% of persons regularly working at least some hours from home did so “to catch up on work.”

2 A comparison of selected characteristics of the sample of employed persons in wave 21 of the HILDA Survey with data from the Population Census is provided in a Supplementary Online Appendix (Table A.1). (Hereafter, numbering for the Online Appendix material will be indicated simply by the “A.” preceding the number.)

3 A dependent child is defined as 1) any household member younger than 15 years; or 2) any household member aged 15 to 24 who is a full-time student, and who is the child of another household member, and who does not have a partner or child of their own in that household.

4 This indicator is derived from a measure of remoteness used by the ABS (2021b). It divides Australia into five remoteness areas based on their relative access to services. We have combined the Outer Regional, Remote, and Very Remote classes.

5 Tables A.2 and A.3 provide a summary of worker characteristics by the extent of working from home in 2019 and 2021.
