# Supplementary material for: Working from Home, COVID-19, and Job Satisfaction
Source: Ind Labor Relat Rev. 2024 Dec 7;78(2):330–54. doi: 10.1177/00197939241301704 (PMC11785509; doi:10.1177/00197939241301704)
Supplement: sj-pdf-1-ilr-10.1177_00197939241301704 – Supplemental material for Working from Home, COVID-19, and Job Satisfaction [file sj-pdf-1-ilr-10.1177_00197939241301704.pdf]

**Online Appendices are the responsibility of the author(s);**
**the material is not copyedited or formatted by the *ILR Review*.**

# Supplementary Online Appendix

# Working from Home, COVID-19, and Job Satisfaction

**Inga Laß, Esperanza Vera-Toscano, and Mark Wooden**

*Table A.1.* Comparison of Selected Characteristics of the HILDA Survey Wave 21 Responding Sample with the 2021 Population Census – Employed Persons (%)

| *Sample / population characteristic* | | *Unweighted HILDA Survey sample* | *Weighted HILDA Survey sample* | *2021 Census* |
|---|---|---|---|---|
| Sex | Male | 50.0 | 52.2 | 51.5 |
| | Female | 50.0 | 47.8 | 48.5 |
| Age group (years) | 15-24 | 14.7 | 14.9 | 14.3 |
| | 25-34 | 24.9 | 23.3 | 22.7 |
| | 35-44 | 20.8 | 22.6 | 22.3 |
| | 45-54 | 19.1 | 20.6 | 20.4 |
| | 55-64 | 15.6 | 14.4 | 15.3 |
| | 65+ | 5.0 | 4.3 | 4.9 |
| Marital status | Married (registered marriage) | 46.1 | 48.2 | 48.4 |
| | Never married | 41.4 | 41.3 | 39.8 |
| | Divorced | 8.4 | 6.8 | 7.7 |
| | Separated | 3.1 | 2.8 | 3.1 |
| | Widowed | 1.0 | 0.9 | 1.0 |
| Aboriginal or Torres Strait Islander | | 3.4 | 2.9 | 2.2 |
| Foreign-born | | 17.6 | 27.5 | 32.1 |
| Speaks language other than English at home | | 9.4 | 18.4 | 23.3 |
| State | New South Wales | 27.8 | 30.8 | 30.5 |
| | Victoria | 26.4 | 27.0 | 26.2 |
| | Queensland | 22.0 | 20.1 | 20.3 |
| | Western Australia | 9.2 | 10.4 | 10.9 |
| | South Australia | 8.2 | 6.9 | 6.9 |
| | Tasmania | 3.4 | 2.2 | 2.1 |
| | Australian Capital Territory | 2.3 | 1.8 | 2.1 |
| | Northern Territory | 0.8 | 0.9 | 1.0 |
| Remoteness | Major city | 67.8 | 75.0 | 73.4 |
| | Inner regional | 31.9 | 17.1 | 16.7 |
| | Outer regional | 9.0 | 7.0 | 7.8 |
| | Remote / Very remote | 1.3 | 1.0 | 2.1 |
| Education attainment | Bachelor's degree or higher | 35.6 | 36.8 | 36.9 |
| | Vocational qualification or Diploma | 34.0 | 32.7 | 32.0 |
| | Year 12 (i.e., completed high school) | 16.6 | 17.7 | 16.6 |

| | | | | |
|---|---|---|---|---|
| | Year 11 or less | 13.8 | 12.8 | 14.5 |
| Occupation (main job) | Managers | 14.2 | 13.8 | 13.9 |
| | Professionals | 26.7 | 25.9 | 24.4 |
| | Technicians & trades workers | 12.5 | 12.4 | 13.1 |
| | Community & personal service workers | 12.0 | 12.5 | 11.7 |
| | Sales workers | 8.0 | 8.3 | 8.3 |
| | Machinery operators & drivers | 6.4 | 7.3 | 6.4 |
| | Labourers | 8.0 | 7.7 | 9.2 |

| | | *Unweighted HILDA Survey sample* | *Weighted HILDA Survey sample* | *2021 Census* |
|---|---|---|---|---|
| Industry (main job) | Agriculture, forestry & fishing | 2.6 | 2.1 | 2.4 |
| | Mining | 2.0 | 2.2 | 1.9 |
| | Manufacturing | 6.9 | 7.1 | 6.2 |
| | Electricity, gas, water & waste services | 1.2 | 1.2 | 1.2 |
| | Construction | 8.6 | 8.4 | 9.3 |
| | Wholesale trade | 2.7 | 2.8 | 2.7 |
| | Retail trade | 8.9 | 8.9 | 9.5 |
| | Accommodation & food services | 5.8 | 5.9 | 6.8 |
| | Transport, postal & warehousing | 3.9 | 4.9 | 4.7 |
| | Information media & telecommunication | 1.3 | 1.4 | 1.4 |
| | Financial & insurance services | 3.5 | 4.2 | 3.9 |
| | Rental, hiring & real estate services | 1.3 | 1.2 | 1.6 |
| | Professional, scientific & technical services | 8.1 | 8.2 | 8.2 |
| | Administrative & support services | 2.9 | 2.7 | 3.4 |
| | Public administration & training | 6.7 | 6.0 | 6.9 |
| | Education & training | 10.4 | 9.9 | 9.2 |
| | Health care & social assistance | 17.9 | 17.6 | 15.2 |
| | Arts & recreation services | 1.8 | 2.0 | 1.6 |
| | Other services | 3.6 | 3.6 | 3.8 |

Notes: 1. All percentages calculated after omitting observations with missing data.

2. Census data extracted using TableBuilder, available on the ABS web site at: https://www.abs.gov.au/statistics/microdata-tablebuilder/tablebuilder

*Table A.2.* Descriptives by Intensity of Working from Home: Females

| | 2019 | | | 2021 | | |
|---|---|---|---|---|---|---|
| *Variable* | *No WFH* | *>0% & <50%* | *>= 50%* | *No WFH* | *>0% & <50%* | *>= 50%* |
| *Age group (years)* | | | | | | |
| 15-24 | 0.108 | 0.018 | 0.018 | 0.068 | 0.020 | 0.018 |
| 25-34 | 0.271 | 0.227 | 0.164 | 0.245 | 0.223 | 0.228 |
| 35-44 | 0.207 | 0.271 | 0.290 | 0.204 | 0.280 | 0.255 |
| 45-54 | 0.214 | 0.285 | 0.220 | 0.218 | 0.247 | 0.260 |
| 55-64 | 0.170 | 0.175 | 0.203 | 0.211 | 0.192 | 0.175 |
| 65 or older | 0.030 | 0.023 | 0.105 | 0.056 | 0.038 | 0.064 |
| *Marital / partnership status* | | | | | | |
| Single | 0.315 | 0.220 | 0.147 | 0.305 | 0.212 | 0.235 |
| Married | 0.472 | 0.602 | 0.664 | 0.491 | 0.594 | 0.571 |
| Cohabiting | 0.213 | 0.178 | 0.189 | 0.205 | 0.194 | 0.194 |
| *Age of youngest child interacted with dependence* | | | | | | |
| Aged 0 to 4 years | 0.140 | 0.150 | 0.220 | 0.135 | 0.165 | 0.156 |
| Aged 5 to 14 years | 0.192 | 0.267 | 0.227 | 0.197 | 0.263 | 0.224 |
| Dependent child aged 15 to 24 years | 0.077 | 0.107 | 0.091 | 0.075 | 0.099 | 0.091 |
| Independent child | 0.073 | 0.050 | 0.063 | 0.084 | 0.062 | 0.051 |
| No children | 0.518 | 0.426 | 0.399 | 0.509 | 0.411 | 0.478 |
| *Health status* | | | | | | |
| Restrictive long-term health condition or disability | 0.090 | 0.095 | 0.150 | 0.110 | 0.106 | 0.107 |
| *Educational attainment* | | | | | | |
| Year 11 and below | 0.126 | 0.033 | 0.119 | 0.140 | 0.062 | 0.057 |
| Year 12 | 0.150 | 0.070 | 0.112 | 0.148 | 0.066 | 0.100 |
| Vocational qualification or Diploma | 0.344 | 0.236 | 0.322 | 0.373 | 0.269 | 0.266 |
| Bachelor's degree or higher | 0.380 | 0.661 | 0.448 | 0.339 | 0.603 | 0.577 |
| *Employment type* | | | | | | |
| Permanent employee | 0.691 | 0.661 | 0.238 | 0.734 | 0.671 | 0.649 |
| Fixed-term contract employee | 0.115 | 0.135 | 0.039 | 0.066 | 0.095 | 0.103 |
| Casual employee | 0.156 | 0.054 | 0.084 | 0.153 | 0.049 | 0.040 |
| Self-employed | 0.036 | 0.146 | 0.622 | 0.045 | 0.181 | 0.203 |
| Other | 0.003 | 0.004 | 0.018 | 0.001 | 0.004 | 0.005 |
| *Tenure with current employer (years)* | | | | | | |
| Less than 1 | 0.190 | 0.117 | 0.094 | 0.161 | 0.143 | 0.121 |
| 1 to <2 | 0.094 | 0.070 | 0.052 | 0.050 | 0.038 | 0.042 |
| 2 to <5 | 0.259 | 0.251 | 0.234 | 0.250 | 0.269 | 0.259 |
| 5 to <10 | 0.203 | 0.212 | 0.227 | 0.234 | 0.210 | 0.244 |
| 10 to <20 | 0.178 | 0.231 | 0.224 | 0.197 | 0.199 | 0.206 |
| 20 or more | 0.076 | 0.118 | 0.168 | 0.108 | 0.141 | 0.127 |
| *Occupation* | | | | | | |
| Managers | 0.086 | 0.222 | 0.203 | 0.077 | 0.221 | 0.171 |
| Professionals | 0.287 | 0.545 | 0.353 | 0.276 | 0.486 | 0.433 |
| Technicians & trades workers | 0.047 | 0.021 | 0.046 | 0.049 | 0.037 | 0.027 |
| Community & personal service workers | 0.183 | 0.050 | 0.077 | 0.208 | 0.070 | 0.059 |
| Clerical & administrative workers | 0.229 | 0.102 | 0.269 | 0.188 | 0.132 | 0.278 |
| Sales workers | 0.090 | 0.040 | 0.042 | 0.104 | 0.035 | 0.028 |
| Machinery operators & drivers | 0.015 | 0.000 | 0.004 | 0.021 | 0.002 | 0.002 |
| Labourers | 0.063 | 0.021 | 0.007 | 0.078 | 0.018 | 0.003 |
| *Other job characteristics* | | | | | | |
| Usual hours worked per week in all jobs | 31.990 | 38.470 | 27.760 | 31.190 | 37.630 | 34.480 |
| Multiple job holder | 0.090 | 0.076 | 0.108 | 0.087 | 0.079 | 0.078 |

| *Variable* | *2019* *No WFH* | *>0% & <50%* | *>= 50%* | *2021* *No WFH* | *>0% & <50%* | *>= 50%* |
|---|---|---|---|---|---|---|
| Normally supervise work of other employees | 0.407 | 0.533 | 0.213 | 0.419 | 0.554 | 0.338 |
| Trade union member | 0.221 | 0.266 | 0.063 | 0.255 | 0.239 | 0.152 |
| Public sector | 0.331 | 0.353 | 0.077 | 0.322 | 0.356 | 0.303 |
| *Firm size (# of employees)* | | | | | | |
| Small (0-19) | 0.174 | 0.225 | 0.745 | 0.196 | 0.269 | 0.300 |
| Medium (20-99) | 0.133 | 0.105 | 0.066 | 0.125 | 0.124 | 0.109 |
| Large (100-499) | 0.124 | 0.123 | 0.032 | 0.113 | 0.119 | 0.118 |
| Very large (500 or more) | 0.513 | 0.533 | 0.133 | 0.484 | 0.450 | 0.442 |
| Firm size unknown | 0.056 | 0.015 | 0.025 | 0.082 | 0.038 | 0.031 |
| *Industry* | | | | | | |
| Agriculture, forestry & fishing | 0.008 | 0.011 | 0.094 | 0.009 | 0.015 | 0.026 |
| Mining | 0.005 | 0.010 | 0.000 | 0.008 | 0.007 | 0.004 |
| Manufacturing | 0.038 | 0.032 | 0.063 | 0.038 | 0.031 | 0.028 |
| Electricity, gas, water & waste services | 0.006 | 0.007 | 0.004 | 0.002 | 0.013 | 0.013 |
| Construction | 0.015 | 0.010 | 0.046 | 0.011 | 0.022 | 0.030 |
| Wholesale trade | 0.017 | 0.025 | 0.025 | 0.016 | 0.017 | 0.025 |
| Retail trade | 0.109 | 0.039 | 0.052 | 0.120 | 0.046 | 0.030 |
| Accommodation & food services | 0.055 | 0.010 | 0.042 | 0.050 | 0.026 | 0.018 |
| Transport, postal & warehousing | 0.024 | 0.018 | 0.018 | 0.024 | 0.007 | 0.021 |
| Information media & telecommunications | 0.011 | 0.017 | 0.011 | 0.006 | 0.007 | 0.025 |
| Financial & insurance services | 0.036 | 0.050 | 0.056 | 0.015 | 0.035 | 0.095 |
| Rental, hiring & real estate services | 0.013 | 0.021 | 0.028 | 0.012 | 0.020 | 0.025 |
| Professional, scientific & technical servs | 0.062 | 0.105 | 0.196 | 0.044 | 0.073 | 0.139 |
| Administrative & support services | 0.030 | 0.033 | 0.035 | 0.026 | 0.027 | 0.031 |
| Public administration & safety | 0.080 | 0.068 | 0.014 | 0.055 | 0.080 | 0.121 |
| Education & training | 0.118 | 0.342 | 0.094 | 0.122 | 0.303 | 0.168 |
| Health care & social assistance | 0.333 | 0.163 | 0.154 | 0.396 | 0.208 | 0.162 |
| Arts & recreation services | 0.011 | 0.017 | 0.028 | 0.014 | 0.017 | 0.014 |
| Other services | 0.031 | 0.028 | 0.042 | 0.028 | 0.046 | 0.030 |
| *Geographical location* | | | | | | |
| Major city | 0.620 | 0.712 | 0.584 | 0.558 | 0.662 | 0.748 |
| Inner regional | 0.267 | 0.204 | 0.280 | 0.295 | 0.250 | 0.203 |
| Outer regional or remote | 0.113 | 0.084 | 0.136 | 0.147 | 0.088 | 0.049 |
| *State* | | | | | | |
| New South Wales | 0.264 | 0.324 | 0.283 | 0.241 | 0.227 | 0.385 |
| Victoria | 0.254 | 0.285 | 0.259 | 0.212 | 0.219 | 0.367 |
| Queensland | 0.224 | 0.179 | 0.255 | 0.245 | 0.272 | 0.131 |
| South Australia | 0.084 | 0.094 | 0.080 | 0.099 | 0.122 | 0.041 |
| Western Australia | 0.098 | 0.065 | 0.105 | 0.117 | 0.101 | 0.041 |
| Tasmania | 0.041 | 0.022 | 0.014 | 0.051 | 0.035 | 0.006 |
| Northern Territory | 0.008 | 0.010 | 0.000 | 0.011 | 0.004 | 0.000 |
| Australian Capital Territory | 0.026 | 0.022 | 0.004 | 0.023 | 0.020 | 0.029 |
| *Interview characteristics* | | | | | | |
| Other adults present during the interview | 0.280 | 0.240 | 0.280 | 0.194 | 0.172 | 0.147 |
| Interviewed by phone | 0.091 | 0.091 | 0.122 | 0.714 | 0.797 | 0.900 |
| Observations | 2558 | 726 | 286 | 2009 | 547 | 1014 |

*Table A.3.* Descriptives by Intensity of Working from Home: Males

| | 2019 | | | 2021 | | |
|---|---|---|---|---|---|---|
| *Variable* | *No WFH* | *>0% & <50%* | *>= 50%* | *No WFH* | *>0% & <50%* | *>= 50%* |
| *Age group (years)* | | | | | | |
| 15-24 | 0.098 | 0.024 | 0.010 | 0.064 | 0.010 | 0.011 |
| 25-34 | 0.298 | 0.219 | 0.123 | 0.268 | 0.191 | 0.217 |
| 35-44 | 0.215 | 0.274 | 0.191 | 0.227 | 0.254 | 0.286 |
| 45-54 | 0.205 | 0.249 | 0.225 | 0.208 | 0.267 | 0.214 |
| 55-64 | 0.155 | 0.192 | 0.255 | 0.186 | 0.221 | 0.179 |
| 65 or older | 0.029 | 0.042 | 0.196 | 0.047 | 0.058 | 0.093 |
| *Marital / partnership status* | | | | | | |
| Single | 0.268 | 0.155 | 0.152 | 0.258 | 0.152 | 0.192 |
| Married | 0.508 | 0.663 | 0.642 | 0.515 | 0.674 | 0.629 |
| Cohabiting | 0.224 | 0.181 | 0.206 | 0.227 | 0.174 | 0.179 |
| *Age of youngest child interacted with dependence* | | | | | | |
| Aged 0 to 4 years | 0.184 | 0.213 | 0.093 | 0.182 | 0.195 | 0.204 |
| Aged 5 to 14 years | 0.179 | 0.226 | 0.225 | 0.172 | 0.243 | 0.203 |
| Dependent child aged 15 to 24 years | 0.053 | 0.097 | 0.074 | 0.059 | 0.083 | 0.080 |
| Independent child | 0.053 | 0.038 | 0.054 | 0.061 | 0.067 | 0.043 |
| No children | 0.531 | 0.426 | 0.554 | 0.526 | 0.412 | 0.470 |
| *Health status* | | | | | | |
| Restrictive long-term health condition or disability | 0.069 | 0.082 | 0.157 | 0.086 | 0.096 | 0.093 |
| *Educational attainment* | | | | | | |
| Year 11 and below | 0.156 | 0.065 | 0.113 | 0.179 | 0.074 | 0.042 |
| Year 12 | 0.163 | 0.094 | 0.113 | 0.166 | 0.094 | 0.100 |
| Vocational qualification or Diploma | 0.426 | 0.379 | 0.348 | 0.462 | 0.430 | 0.278 |
| Bachelor’s degree or higher | 0.255 | 0.462 | 0.426 | 0.192 | 0.402 | 0.580 |
| *Employment type* | | | | | | |
| Permanent employee | 0.703 | 0.541 | 0.240 | 0.715 | 0.515 | 0.650 |
| Fixed-term contract employee | 0.077 | 0.077 | 0.025 | 0.045 | 0.052 | 0.070 |
| Casual employee | 0.134 | 0.024 | 0.015 | 0.127 | 0.017 | 0.012 |
| Self-employed | 0.084 | 0.358 | 0.701 | 0.111 | 0.414 | 0.263 |
| Other | 0.001 | 0.001 | 0.020 | 0.001 | 0.001 | 0.005 |
| *Tenure with current employer (years)* | | | | | | |
| Less than 1 | 0.167 | 0.112 | 0.059 | 0.151 | 0.080 | 0.115 |
| 1 to <2 | 0.092 | 0.073 | 0.044 | 0.063 | 0.034 | 0.060 |
| 2 to <5 | 0.251 | 0.234 | 0.186 | 0.258 | 0.222 | 0.223 |
| 5 to <10 | 0.205 | 0.213 | 0.206 | 0.214 | 0.228 | 0.230 |
| 10 to <20 | 0.188 | 0.217 | 0.216 | 0.199 | 0.253 | 0.218 |
| 20 or more | 0.097 | 0.151 | 0.289 | 0.116 | 0.184 | 0.154 |
| *Occupation* | | | | | | |
| Managers | 0.144 | 0.311 | 0.407 | 0.127 | 0.295 | 0.323 |
| Professionals | 0.175 | 0.350 | 0.373 | 0.123 | 0.289 | 0.463 |
| Technicians & trades workers | 0.236 | 0.158 | 0.118 | 0.258 | 0.212 | 0.075 |
| Community & personal service workers | 0.079 | 0.037 | 0.010 | 0.084 | 0.039 | 0.020 |
| Clerical & administrative workers | 0.067 | 0.046 | 0.049 | 0.063 | 0.038 | 0.072 |
| Sales workers | 0.048 | 0.024 | 0.010 | 0.048 | 0.025 | 0.036 |
| Machinery operators & drivers | 0.141 | 0.029 | 0.010 | 0.172 | 0.045 | 0.004 |
| Labourers | 0.110 | 0.046 | 0.025 | 0.126 | 0.056 | 0.009 |
| *Other job characteristics* | | | | | | |
| Usual hours worked per week in all jobs | 41.450 | 44.590 | 40.030 | 40.890 | 44.400 | 39.490 |
| Multiple job holder | 0.070 | 0.060 | 0.064 | 0.061 | 0.070 | 0.072 |

| Variable | 2019 No WFH | 2019 >0% & <50% | 2019 >= 50% | 2021 No WFH | 2021 >0% & <50% | 2021 >= 50% |
|---|---|---|---|---|---|---|
| Normally supervise work of other employees | 0.523 | 0.589 | 0.314 | 0.504 | 0.574 | 0.453 |
| Trade union member | 0.190 | 0.106 | 0.029 | 0.201 | 0.124 | 0.075 |
| Public sector | 0.196 | 0.164 | 0.034 | 0.175 | 0.170 | 0.169 |
| *Firm size (# of employees)* | | | | | | |
| Small (0-19) | 0.246 | 0.424 | 0.784 | 0.281 | 0.471 | 0.324 |
| Medium (20-99) | 0.161 | 0.092 | 0.054 | 0.158 | 0.100 | 0.122 |
| Large (100-499) | 0.126 | 0.105 | 0.029 | 0.120 | 0.107 | 0.142 |
| Very large (500 or more) | 0.435 | 0.367 | 0.118 | 0.389 | 0.313 | 0.395 |
| Firm size unknown | 0.031 | 0.012 | 0.015 | 0.052 | 0.010 | 0.016 |
| *Industry* | | | | | | |
| Agriculture, forestry & fishing | 0.026 | 0.037 | 0.196 | 0.032 | 0.045 | 0.056 |
| Mining | 0.043 | 0.018 | 0.000 | 0.048 | 0.021 | 0.009 |
| Manufacturing | 0.131 | 0.068 | 0.083 | 0.139 | 0.073 | 0.056 |
| Electricity, gas, water & waste services | 0.021 | 0.017 | 0.005 | 0.022 | 0.014 | 0.012 |
| Construction | 0.150 | 0.170 | 0.025 | 0.172 | 0.218 | 0.048 |
| Wholesale trade | 0.042 | 0.045 | 0.044 | 0.041 | 0.044 | 0.051 |
| Retail trade | 0.074 | 0.028 | 0.029 | 0.078 | 0.025 | 0.031 |
| Accommodation & food services | 0.034 | 0.017 | 0.034 | 0.031 | 0.018 | 0.012 |
| Transport, postal & warehousing | 0.079 | 0.038 | 0.029 | 0.092 | 0.034 | 0.011 |
| Information media & telecommunications | 0.009 | 0.021 | 0.029 | 0.005 | 0.010 | 0.040 |
| Financial & insurance services | 0.025 | 0.048 | 0.083 | 0.008 | 0.024 | 0.126 |
| Rental, hiring & real estate services | 0.012 | 0.020 | 0.005 | 0.009 | 0.018 | 0.017 |
| Professional, scientific & technical servs | 0.059 | 0.150 | 0.245 | 0.037 | 0.118 | 0.247 |
| Administrative & support services | 0.026 | 0.041 | 0.010 | 0.030 | 0.044 | 0.018 |
| Public administration & safety | 0.086 | 0.070 | 0.010 | 0.071 | 0.079 | 0.085 |
| Education & training | 0.040 | 0.122 | 0.049 | 0.036 | 0.093 | 0.085 |
| Health care & social assistance | 0.087 | 0.044 | 0.015 | 0.090 | 0.072 | 0.045 |
| Arts & recreation services | 0.018 | 0.017 | 0.029 | 0.019 | 0.020 | 0.017 |
| Other services | 0.038 | 0.032 | 0.078 | 0.042 | 0.032 | 0.033 |
| *Geographical location* | | | | | | |
| Major city | 0.615 | 0.698 | 0.574 | 0.557 | 0.636 | 0.780 |
| Inner regional | 0.267 | 0.223 | 0.255 | 0.303 | 0.270 | 0.165 |
| Outer regional or remote | 0.118 | 0.079 | 0.172 | 0.140 | 0.094 | 0.055 |
| *State* | | | | | | |
| New South Wales | 0.271 | 0.320 | 0.289 | 0.253 | 0.285 | 0.367 |
| Victoria | 0.259 | 0.250 | 0.304 | 0.231 | 0.202 | 0.383 |
| Queensland | 0.228 | 0.201 | 0.186 | 0.256 | 0.226 | 0.120 |
| South Australia | 0.080 | 0.092 | 0.069 | 0.092 | 0.112 | 0.036 |
| Western Australia | 0.095 | 0.071 | 0.108 | 0.106 | 0.100 | 0.039 |
| Tasmania | 0.035 | 0.034 | 0.020 | 0.041 | 0.041 | 0.011 |
| Northern Territory | 0.009 | 0.006 | 0.005 | 0.008 | 0.006 | 0.002 |
| Australian Capital Territory | 0.023 | 0.026 | 0.020 | 0.013 | 0.028 | 0.042 |
| *Interview characteristics* | | | | | | |
| Other adults present during the interview | 0.340 | 0.312 | 0.299 | 0.223 | 0.230 | 0.144 |
| Interviewed by phone | 0.096 | 0.102 | 0.103 | 0.720 | 0.779 | 0.900 |
| Observations | 2744 | 849 | 204 | 2268 | 712 | 817 |

*Table A.4.* Working from Home and Job Satisfaction (Fixed Effects Regression Results): Females

| Variable | Any hours from home (1) | Most hours from home (2) | WFH categories (3) | Proportion from home (4) | Proportion from home plus its square (5) |
|---|---|---|---|---|---|
| *Working from home* | | | | | |
| Any hours worked from home | 0.220*** | | | | |
| | (0.060) | | | | |
| 50% or more of hours worked from home | | 0.247*** | | | |
| | | (0.064) | | | |
| Proportion of hours worked from home | | | | 0.236** | 1.365*** |
| | | | | (0.073) | (0.355) |
| Proportion of hours worked from home squared | | | | | -1.137** |
| | | | | | (0.348) |
| Share of hours worked from home (%) (ref. group = 0) | | | | | |
| 1-19 | | | 0.129 | | |
| | | | (0.080) | | |
| 20-39 | | | 0.201* | | |
| | | | (0.094) | | |
| 40-59 | | | 0.314* | | |
| | | | (0.137) | | |
| 60-79 | | | 0.607*** | | |
| | | | (0.178) | | |
| 80-99 | | | 0.382* | | |
| | | | (0.156) | | |
| 100 | | | 0.202** | | |
| | | | (0.076) | | |
| *Year = 2021* | 0.003 | -0.000 | -0.001 | 0.000 | -0.004 |
| | (0.053) | (0.053) | (0.053) | (0.053) | (0.053) |
| *Age group (ref. group = 35-44 years)* | | | | | |
| 15-24 years | -0.251 | -0.271 | -0.256 | -0.267 | -0.246 |
| | (0.202) | (0.201) | (0.202) | (0.202) | (0.201) |
| 25-34 years | -0.175 | -0.187 | -0.178 | -0.183 | -0.170 |
| | (0.122) | (0.122) | (0.121) | (0.122) | (0.121) |
| 45-54 years | 0.084 | 0.076 | 0.088 | 0.076 | 0.084 |
| | (0.123) | (0.123) | (0.124) | (0.123) | (0.123) |
| 55-64 years | -0.064 | -0.081 | -0.062 | -0.084 | -0.068 |
| | (0.192) | (0.192) | (0.192) | (0.192) | (0.191) |
| 65 years or older | 0.175 | 0.139 | 0.175 | 0.140 | 0.169 |
| | (0.261) | (0.261) | (0.262) | (0.261) | (0.261) |
| *Marital / partnering status (ref. group = Single)* | | | | | |
| Married | -0.049 | -0.049 | -0.061 | -0.047 | -0.056 |
| | (0.166) | (0.165) | (0.166) | (0.166) | (0.165) |
| Cohabiting | -0.043 | -0.033 | -0.049 | -0.036 | -0.047 |
| | (0.132) | (0.132) | (0.132) | (0.132) | (0.132) |
| *Age of youngest child interacted with dependence (ref. group = No children)* | | | | | |
| Aged 0 to 4 years | 0.160 | 0.174 | 0.156 | 0.168 | 0.147 |
| | (0.138) | (0.139) | (0.137) | (0.139) | (0.137) |
| Aged 5 to 14 years | 0.271 | 0.271 | 0.258 | 0.272 | 0.257 |
| | (0.146) | (0.147) | (0.146) | (0.147) | (0.146) |

| Variable | Any hours from home (1) | Most hours from home (2) | WFH categories (3) | Proportion from home (4) | Proportion from home plus its square (5) |
|---|---|---|---|---|---|
| Dependent child aged 15 to 24 years | 0.102 | 0.094 | 0.100 | 0.097 | 0.100 |
| | (0.130) | (0.131) | (0.130) | (0.131) | (0.130) |
| Independent child | -0.146 | -0.155 | -0.142 | -0.153 | -0.148 |
| | (0.129) | (0.129) | (0.129) | (0.129) | (0.128) |
| *Health status* | | | | | |
| Has restrictive long-term health condition or disability | -0.094 | -0.088 | -0.088 | -0.092 | -0.093 |
| | (0.107) | (0.107) | (0.107) | (0.107) | (0.107) |
| *Educational attainment (ref. group = Year 11 and below)* | | | | | |
| Year 12 | 0.237 | 0.204 | 0.215 | 0.217 | 0.208 |
| | (0.739) | (0.741) | (0.736) | (0.747) | (0.730) |
| Vocational qual. or diploma | 0.827 | 0.823 | 0.823 | 0.832 | 0.808 |
| | (0.613) | (0.612) | (0.607) | (0.620) | (0.601) |
| Bachelor's degree or higher | 1.514* | 1.495* | 1.483* | 1.508* | 1.468* |
| | (0.760) | (0.761) | (0.755) | (0.768) | (0.748) |
| *Employment type (ref. group = Permanent employee)* | | | | | |
| Fixed-term contract employee | 0.144 | 0.139 | 0.149 | 0.139 | 0.146 |
| | (0.096) | (0.096) | (0.096) | (0.096) | (0.096) |
| Casual employee | -0.053 | -0.053 | -0.051 | -0.054 | -0.057 |
| | (0.118) | (0.118) | (0.118) | (0.118) | (0.118) |
| Self-employed | 0.201 | 0.209 | 0.195 | 0.211 | 0.191 |
| | (0.167) | (0.166) | (0.167) | (0.167) | (0.167) |
| Other | 0.877* | 0.888* | 0.863* | 0.890* | 0.846* |
| | (0.350) | (0.349) | (0.350) | (0.350) | (0.352) |
| *Tenure with current employer (ref. group = <1 year)* | | | | | |
| 1 year to less than 2 years | -0.014 | -0.013 | -0.016 | -0.015 | -0.018 |
| | (0.105) | (0.105) | (0.105) | (0.105) | (0.105) |
| 2 to < 5 years | -0.242** | -0.239** | -0.242** | -0.240** | -0.244** |
| | (0.076) | (0.076) | (0.076) | (0.076) | (0.076) |
| 5 to < 10 years | -0.414*** | -0.419*** | -0.415*** | -0.418*** | -0.419*** |
| | (0.091) | (0.091) | (0.091) | (0.092) | (0.091) |
| 10 to < 20 years | -0.372** | -0.371** | -0.369** | -0.370** | -0.378** |
| | (0.117) | (0.116) | (0.117) | (0.117) | (0.116) |
| 20 or more years | -0.385* | -0.397** | -0.381* | -0.394** | -0.392** |
| | (0.150) | (0.150) | (0.151) | (0.150) | (0.151) |
| *Occupation (ref. group = Labourers)* | | | | | |
| Managers | 0.008 | 0.017 | 0.027 | 0.015 | 0.018 |
| | (0.248) | (0.248) | (0.248) | (0.248) | (0.248) |
| Professionals | 0.215 | 0.234 | 0.222 | 0.231 | 0.215 |
| | (0.248) | (0.248) | (0.248) | (0.248) | (0.248) |
| Technicians & trades workers | 0.355 | 0.362 | 0.346 | 0.360 | 0.349 |
| | (0.299) | (0.298) | (0.299) | (0.298) | (0.299) |
| Community & personal service | -0.056 | -0.056 | -0.051 | -0.057 | -0.048 |
| | (0.249) | (0.249) | (0.250) | (0.249) | (0.250) |
| Clerical & administrative workers | 0.175 | 0.183 | 0.187 | 0.181 | 0.182 |
| | (0.253) | (0.253) | (0.253) | (0.253) | (0.253) |
| Sales workers | 0.007 | 0.012 | 0.009 | 0.013 | 0.010 |
| | (0.290) | (0.290) | (0.290) | (0.290) | (0.290) |
| Machinery operators & drivers | -0.056 | -0.053 | -0.060 | -0.053 | -0.051 |
| | (0.349) | (0.348) | (0.350) | (0.348) | (0.350) |
| *Other job characteristics* | | | | | |
| Usual hours worked per week | 0.022* | 0.022* | 0.022* | 0.022* | 0.022* |
| | (0.009) | (0.009) | (0.009) | (0.009) | (0.009) |

| Variable | Any hours from home (1) | Most hours from home (2) | WFH categories (3) | Proportion from home (4) | Proportion from home plus its square (5) |
|---|---|---|---|---|---|
| Usual hours worked per week (squared) | -0.000** | -0.000* | -0.000* | -0.000* | -0.000* |
| | (0.000) | (0.000) | (0.000) | (0.000) | (0.000) |
| Multiple job holder | -0.021 | -0.021 | -0.020 | -0.023 | -0.015 |
| | (0.107) | (0.106) | (0.107) | (0.106) | (0.106) |
| Supervisor | -0.055 | -0.046 | -0.055 | -0.050 | -0.055 |
| | (0.063) | (0.063) | (0.063) | (0.063) | (0.063) |
| Trade union member | -0.286* | -0.279* | -0.272* | -0.282* | -0.277* |
| | (0.114) | (0.114) | (0.115) | (0.115) | (0.114) |
| Public sector | 0.081 | 0.088 | 0.085 | 0.087 | 0.084 |
| | (0.109) | (0.109) | (0.109) | (0.109) | (0.109) |
| *Firm size (ref. group = Small (0-19 employees))* | | | | | |
| Medium (20-99 employees) | -0.118 | -0.124 | -0.124 | -0.120 | -0.122 |
| | (0.139) | (0.138) | (0.139) | (0.139) | (0.139) |
| Large (100-499 employees) | -0.096 | -0.104 | -0.104 | -0.102 | -0.102 |
| | (0.141) | (0.140) | (0.141) | (0.140) | (0.140) |
| Very large (500 or more) | -0.207 | -0.214 | -0.208 | -0.215 | -0.207 |
| | (0.139) | (0.139) | (0.139) | (0.139) | (0.139) |
| Firm size unknown | -0.066 | -0.077 | -0.067 | -0.075 | -0.069 |
| | (0.158) | (0.158) | (0.158) | (0.158) | (0.158) |
| *Industry (ref. group = Professional, scientific & technical services)* | | | | | |
| Agriculture, forestry & fishing | -0.129 | -0.160 | -0.121 | -0.149 | -0.130 |
| | (0.421) | (0.424) | (0.424) | (0.424) | (0.425) |
| Mining | 0.167 | 0.187 | 0.217 | 0.171 | 0.173 |
| | (0.557) | (0.553) | (0.566) | (0.550) | (0.561) |
| Manufacturing | 0.006 | -0.013 | 0.015 | -0.021 | -0.006 |
| | (0.310) | (0.310) | (0.311) | (0.310) | (0.309) |
| Electricity, gas, water & waste | 0.565 | 0.616 | 0.565 | 0.604 | 0.538 |
| | (0.433) | (0.420) | (0.438) | (0.425) | (0.445) |
| Construction | 0.441 | 0.401 | 0.413 | 0.405 | 0.419 |
| | (0.370) | (0.367) | (0.366) | (0.368) | (0.367) |
| Wholesale trade | -0.053 | -0.063 | -0.050 | -0.065 | -0.059 |
| | (0.283) | (0.285) | (0.280) | (0.285) | (0.281) |
| Retail trade | 0.062 | 0.040 | 0.052 | 0.041 | 0.046 |
| | (0.256) | (0.256) | (0.257) | (0.256) | (0.256) |
| Accommodation & food services | 0.098 | 0.086 | 0.106 | 0.080 | 0.095 |
| | (0.288) | (0.287) | (0.289) | (0.287) | (0.289) |
| Transport, postal & warehousing | 0.023 | -0.002 | 0.013 | 0.003 | 0.006 |
| | (0.298) | (0.296) | (0.300) | (0.296) | (0.298) |
| Information media, communication | 0.533 | 0.515 | 0.574 | 0.509 | 0.555 |
| | (0.365) | (0.366) | (0.371) | (0.364) | (0.368) |
| Financial & insurance services | 0.086 | 0.048 | 0.061 | 0.057 | 0.063 |
| | (0.318) | (0.316) | (0.314) | (0.318) | (0.316) |
| Rental, hiring, real estate services | -0.022 | -0.012 | -0.038 | -0.013 | -0.017 |
| | (0.413) | (0.407) | (0.406) | (0.409) | (0.406) |
| Administrative & support services | 0.038 | 0.049 | 0.051 | 0.040 | 0.042 |
| | (0.234) | (0.236) | (0.234) | (0.235) | (0.234) |
| Public administration & safety | 0.608** | 0.610** | 0.612** | 0.604** | 0.604** |
| | (0.232) | (0.232) | (0.233) | (0.232) | (0.232) |
| Education & training | 0.282 | 0.292 | 0.276 | 0.285 | 0.273 |
| | (0.238) | (0.237) | (0.239) | (0.237) | (0.238) |
| Health care & social assistance | 0.529** | 0.525** | 0.529** | 0.517* | 0.524** |
| | (0.203) | (0.202) | (0.203) | (0.202) | (0.203) |

| Variable | Any hours from home (1) | Most hours from home (2) | WFH categories (3) | Proportion from home (4) | Proportion from home plus its square (5) |
|---|---|---|---|---|---|
| Arts & recreation services | 0.798** | 0.794** | 0.814** | 0.783** | 0.790** |
| | (0.275) | (0.270) | (0.273) | (0.271) | (0.274) |
| Other services | 0.191 | 0.151 | 0.217 | 0.151 | 0.200 |
| | (0.254) | (0.256) | (0.257) | (0.255) | (0.256) |
| *Geographical location (ref. group = Outer regional or remote)* | | | | | |
| Major city | -0.098 | -0.098 | -0.086 | -0.099 | -0.097 |
| | (0.262) | (0.264) | (0.263) | (0.263) | (0.262) |
| Inner regional | 0.052 | 0.046 | 0.065 | 0.047 | 0.055 |
| | (0.229) | (0.231) | (0.232) | (0.230) | (0.229) |
| *State (ref. group = Victoria (VIC))* | | | | | |
| New South Wales (NSW) | 0.016 | 0.019 | 0.002 | 0.024 | 0.017 |
| | (0.353) | (0.355) | (0.360) | (0.354) | (0.358) |
| Queensland (QLD) | -0.281 | -0.288 | -0.297 | -0.284 | -0.289 |
| | (0.337) | (0.340) | (0.342) | (0.339) | (0.340) |
| South Australia (SA) | 0.171 | 0.196 | 0.221 | 0.185 | 0.210 |
| | (0.621) | (0.608) | (0.616) | (0.610) | (0.611) |
| Western Australia (WA) | -0.034 | -0.022 | -0.038 | -0.006 | -0.020 |
| | (0.930) | (0.912) | (0.928) | (0.917) | (0.929) |
| Tasmania (TAS) | -0.590 | -0.606 | -0.574 | -0.613 | -0.551 |
| | (0.645) | (0.639) | (0.644) | (0.647) | (0.641) |
| Northern Territory (NT) | 2.679*** | 2.675*** | 2.885*** | 2.629*** | 2.773*** |
| | (0.591) | (0.589) | (0.671) | (0.570) | (0.623) |
| Australian Capital Territory (ACT) | -0.114 | -0.112 | -0.097 | -0.123 | -0.104 |
| | (0.539) | (0.546) | (0.542) | (0.544) | (0.546) |
| *Interview characteristics* | | | | | |
| Other adults present during i'view | -0.055 | -0.055 | -0.054 | -0.056 | -0.057 |
| | (0.058) | (0.058) | (0.058) | (0.058) | (0.058) |
| Interviewed by phone | 0.128* | 0.109 | 0.117* | 0.116 | 0.119* |
| | (0.059) | (0.059) | (0.059) | (0.059) | (0.060) |
| Constant | 6.554*** | 6.593*** | 6.559*** | 6.587*** | 6.582*** |
| | (0.753) | (0.754) | (0.750) | (0.759) | (0.745) |
| R-squared | 0.056 | 0.056 | 0.059 | 0.055 | 0.058 |
| Rho | 0.616 | 0.616 | 0.616 | 0.615 | 0.616 |
| Observations | 7140 | 7140 | 7140 | 7140 | 7140 |

*Notes*: WFH = Working from home.
*$p$<0.05, **$p$<0.01, ***$p$<0.001.

*Table A.5*. Working from Home and Job Satisfaction (Fixed Effects Regression Results): Males

| Variable | Any hours from home (1) | Most hours from home (2) | WFH categories (3) | Proportion from home (4) | Proportion from home plus its square (5) |
|---|---|---|---|---|---|
| *Working from home* | | | | | |
| Any hours worked from home | 0.069 | | | | |
| | (0.053) | | | | |
| 50% or more of hours worked from home | | 0.059 | | | |
| | | (0.063) | | | |
| Proportion of hours worked from home | | | | 0.037 | 0.043 |
| | | | | (0.074) | (0.317) |
| Proportion of hours worked from home squared | | | | | -0.005 |
| | | | | | (0.310) |
| Share of hours worked from home (%) (ref. group = 0) | | | | | |
| 1-19 | | | 0.111 | | |
| | | | (0.064) | | |
| 20-39 | | | -0.085 | | |
| | | | (0.088) | | |
| 40-59 | | | 0.170 | | |
| | | | (0.112) | | |
| 60-79 | | | 0.213 | | |
| | | | (0.128) | | |
| 80-99 | | | 0.085 | | |
| | | | (0.184) | | |
| 100 | | | 0.043 | | |
| | | | (0.079) | | |
| *Year = 2021* | 0.106* | 0.106* | 0.106* | 0.107* | 0.107* |
| | (0.045) | (0.046) | (0.046) | (0.046) | (0.046) |
| *Age group (ref. group = 35-44* years*)* | | | | | |
| 15-24 years | -0.064 | -0.072 | -0.061 | -0.073 | -0.073 |
| | (0.182) | (0.182) | (0.183) | (0.182) | (0.182) |
| 25-34 years | -0.035 | -0.038 | -0.032 | -0.039 | -0.039 |
| | (0.100) | (0.100) | (0.100) | (0.100) | (0.100) |
| 45-54 years | 0.023 | 0.026 | 0.027 | 0.027 | 0.027 |
| | (0.119) | (0.118) | (0.119) | (0.119) | (0.119) |
| 55-64 years | -0.114 | -0.111 | -0.106 | -0.110 | -0.111 |
| | (0.190) | (0.190) | (0.191) | (0.190) | (0.190) |
| 65 years or older | -0.141 | -0.138 | -0.129 | -0.139 | -0.139 |
| | (0.274) | (0.274) | (0.274) | (0.274) | (0.274) |
| *Marital / partnering status (ref. group = Single)* | | | | | |
| Married | 0.018 | 0.016 | 0.021 | 0.015 | 0.015 |
| | (0.143) | (0.143) | (0.143) | (0.143) | (0.143) |
| Cohabiting | 0.103 | 0.104 | 0.106 | 0.103 | 0.103 |
| | (0.114) | (0.114) | (0.115) | (0.114) | (0.114) |
| *Age of youngest child interacted with dependence (ref. group = No children)* | | | | | |
| Aged 0 to 4 years | -0.117 | -0.120 | -0.126 | -0.117 | -0.117 |
| | (0.099) | (0.099) | (0.099) | (0.099) | (0.099) |
| Aged 5 to 14 years | 0.101 | 0.098 | 0.094 | 0.101 | 0.101 |
| | (0.111) | (0.111) | (0.111) | (0.111) | (0.111) |

| Variable | Any hours from home (1) | Most hours from home (2) | WFH categories (3) | Proportion from home (4) | Proportion from home plus its square (5) |
|---|---|---|---|---|---|
| Dependent child aged 15 to 24 years | 0.099 | 0.094 | 0.085 | 0.096 | 0.096 |
| | (0.126) | (0.126) | (0.126) | (0.126) | (0.126) |
| Independent child | 0.097 | 0.091 | 0.082 | 0.092 | 0.092 |
| | (0.114) | (0.114) | (0.114) | (0.114) | (0.114) |
| *Health status* | | | | | |
| Has restrictive long-term health condition or disability | -0.037 | -0.036 | -0.038 | -0.036 | -0.036 |
| | (0.095) | (0.095) | (0.095) | (0.095) | (0.095) |
| *Educational attainment (ref. group = Year 11 and below)* | | | | | |
| Year 12 | 0.699 | 0.679 | 0.669 | 0.681 | 0.681 |
| | (0.810) | (0.808) | (0.803) | (0.809) | (0.808) |
| Vocational qual. or diploma | 1.011 | 0.997 | 0.984 | 0.994 | 0.994 |
| | (0.692) | (0.690) | (0.684) | (0.691) | (0.690) |
| Bachelor's degree or higher | 0.701 | 0.689 | 0.675 | 0.692 | 0.692 |
| | (0.774) | (0.771) | (0.766) | (0.773) | (0.773) |
| *Employment type (ref. group = Permanent employee)* | | | | | |
| Fixed-term contract employee | -0.220* | -0.217* | -0.223* | -0.217* | -0.217* |
| | (0.090) | (0.090) | (0.090) | (0.090) | (0.090) |
| Casual employee | -0.110 | -0.113 | -0.113 | -0.113 | -0.113 |
| | (0.118) | (0.118) | (0.118) | (0.118) | (0.118) |
| Self-employed | 0.081 | 0.096 | 0.079 | 0.095 | 0.095 |
| | (0.116) | (0.115) | (0.117) | (0.115) | (0.116) |
| Other | 0.473 | 0.480 | 0.454 | 0.485 | 0.485 |
| | (0.324) | (0.330) | (0.327) | (0.329) | (0.329) |
| *Tenure with current employer (ref. group =<1 year)* | | | | | |
| 1 year to less than 2 years | -0.099 | -0.097 | -0.095 | -0.096 | -0.096 |
| | (0.094) | (0.094) | (0.094) | (0.094) | (0.094) |
| 2 to < 5 years | -0.226*** | -0.225*** | -0.222*** | -0.226*** | -0.226*** |
| | (0.067) | (0.067) | (0.067) | (0.067) | (0.067) |
| 5 to < 10 years | -0.495*** | -0.495*** | -0.497*** | -0.495*** | -0.495*** |
| | (0.083) | (0.083) | (0.083) | (0.083) | (0.083) |
| 10 to < 20 years | -0.538*** | -0.538*** | -0.545*** | -0.537*** | -0.537*** |
| | (0.103) | (0.103) | (0.103) | (0.103) | (0.103) |
| 20 or more years | -0.687*** | -0.686*** | -0.693*** | -0.685*** | -0.685*** |
| | (0.144) | (0.144) | (0.144) | (0.144) | (0.144) |
| *Occupation (ref. group = Labourers)* | | | | | |
| Managers | 0.093 | 0.098 | 0.096 | 0.099 | 0.099 |
| | (0.150) | (0.150) | (0.149) | (0.150) | (0.149) |
| Professionals | 0.091 | 0.096 | 0.103 | 0.095 | 0.095 |
| | (0.164) | (0.164) | (0.164) | (0.165) | (0.164) |
| Technicians & trades workers | 0.116 | 0.116 | 0.108 | 0.116 | 0.116 |
| | (0.156) | (0.156) | (0.156) | (0.156) | (0.156) |
| Community & personal service | 0.511* | 0.511* | 0.507* | 0.511* | 0.511* |
| | (0.258) | (0.259) | (0.258) | (0.259) | (0.259) |
| Clerical & administrative workers | 0.072 | 0.076 | 0.070 | 0.076 | 0.075 |
| | (0.194) | (0.194) | (0.194) | (0.194) | (0.195) |
| Sales workers | 0.156 | 0.156 | 0.154 | 0.158 | 0.158 |
| | (0.202) | (0.202) | (0.202) | (0.202) | (0.202) |
| Machinery operators & drivers | 0.104 | 0.102 | 0.104 | 0.101 | 0.101 |
| | (0.164) | (0.164) | (0.164) | (0.164) | (0.164) |
| *Other job characteristics* | | | | | |
| Usual hours worked per week | 0.017 | 0.017 | 0.017 | 0.017 | 0.017 |
| | (0.011) | (0.011) | (0.011) | (0.011) | (0.011) |

| Variable | Any hours from home (1) | Most hours from home (2) | WFH categories (3) | Proportion from home (4) | Proportion from home plus its square (5) |
|---|---|---|---|---|---|
| Usual hours worked per week (squared) | -0.000 | -0.000 | -0.000 | -0.000 | -0.000 |
| | (0.000) | (0.000) | (0.000) | (0.000) | (0.000) |
| Multiple job holder | -0.024 | -0.024 | -0.022 | -0.024 | -0.024 |
| | (0.114) | (0.114) | (0.114) | (0.114) | (0.114) |
| Supervisor | -0.064 | -0.064 | -0.064 | -0.064 | -0.064 |
| | (0.053) | (0.053) | (0.053) | (0.053) | (0.053) |
| Trade union member | -0.135 | -0.136 | -0.135 | -0.136 | -0.136 |
| | (0.113) | (0.113) | (0.113) | (0.113) | (0.113) |
| Public sector | 0.051 | 0.047 | 0.051 | 0.048 | 0.048 |
| | (0.157) | (0.157) | (0.156) | (0.157) | (0.157) |
| *Firm size (ref. group = Small (0-19 employees))* | | | | | |
| Medium (20-99 employees) | -0.008 | -0.011 | -0.011 | -0.010 | -0.010 |
| | (0.098) | (0.098) | (0.098) | (0.098) | (0.098) |
| Large (100-499 employees) | -0.039 | -0.043 | -0.040 | -0.042 | -0.042 |
| | (0.116) | (0.116) | (0.116) | (0.116) | (0.116) |
| Very large (500 or more) | 0.084 | 0.081 | 0.080 | 0.081 | 0.081 |
| | (0.116) | (0.116) | (0.116) | (0.116) | (0.116) |
| Firm size unknown | 0.132 | 0.130 | 0.121 | 0.130 | 0.130 |
| | (0.166) | (0.166) | (0.166) | (0.166) | (0.166) |
| *Industry (ref. group* = Professional, scientific & technical services) | | | | | |
| Agriculture, forestry & fishing | -0.513* | -0.517* | -0.515* | -0.515* | -0.515* |
| | (0.215) | (0.216) | (0.215) | (0.216) | (0.216) |
| Mining | -0.082 | -0.083 | -0.086 | -0.085 | -0.085 |
| | (0.234) | (0.235) | (0.232) | (0.234) | (0.235) |
| Manufacturing | -0.301 | -0.302 | -0.302 | -0.303 | -0.303 |
| | (0.172) | (0.173) | (0.173) | (0.173) | (0.173) |
| Electricity, gas, water & waste | -0.320 | -0.313 | -0.318 | -0.314 | -0.314 |
| | (0.275) | (0.275) | (0.277) | (0.275) | (0.275) |
| Construction | -0.421** | -0.423** | -0.425** | -0.424** | -0.424** |
| | (0.154) | (0.154) | (0.153) | (0.154) | (0.154) |
| Wholesale trade | -0.411* | -0.409* | -0.415* | -0.408* | -0.408* |
| | (0.183) | (0.182) | (0.182) | (0.182) | (0.182) |
| Retail trade | -0.523* | -0.528* | -0.529* | -0.527* | -0.527* |
| | (0.237) | (0.238) | (0.237) | (0.238) | (0.238) |
| Accommodation & food services | -1.418*** | -1.422*** | -1.434*** | -1.423*** | -1.423*** |
| | (0.335) | (0.336) | (0.336) | (0.336) | (0.336) |
| Transport, postal & warehousing | -0.451* | -0.453* | -0.459* | -0.453* | -0.453* |
| | (0.205) | (0.205) | (0.204) | (0.205) | (0.205) |
| Information media, communication | 0.534 | 0.535 | 0.543 | 0.531 | 0.531 |
| | (0.354) | (0.355) | (0.352) | (0.355) | (0.355) |
| Financial & insurance services | -0.085 | -0.085 | -0.083 | -0.082 | -0.083 |
| | (0.238) | (0.238) | (0.239) | (0.238) | (0.238) |
| Rental, hiring, real estate services | -0.206 | -0.205 | -0.214 | -0.208 | -0.208 |
| | (0.328) | (0.328) | (0.331) | (0.329) | (0.329) |
| Administrative & support services | -0.126 | -0.123 | -0.133 | -0.124 | -0.124 |
| | (0.209) | (0.210) | (0.210) | (0.209) | (0.210) |
| Public administration & safety | -0.349 | -0.347 | -0.352 | -0.351 | -0.351 |
| | (0.200) | (0.201) | (0.201) | (0.200) | (0.201) |
| Education & training | -0.009 | -0.001 | -0.003 | -0.004 | -0.004 |
| | (0.262) | (0.263) | (0.265) | (0.263) | (0.264) |
| Health care & social assistance | -0.256 | -0.254 | -0.261 | -0.255 | -0.255 |
| | (0.223) | (0.223) | (0.224) | (0.223) | (0.223) |

| Variable | Any hours from home (1) | Most hours from home (2) | WFH categories (3) | Proportion from home (4) | Proportion from home plus its square (5) |
|---|---|---|---|---|---|
| Arts & recreation services | -0.112 | -0.109 | -0.127 | -0.110 | -0.110 |
| | (0.286) | (0.287) | (0.286) | (0.287) | (0.287) |
| Other services | -0.551** | -0.553** | -0.565** | -0.551** | -0.551** |
| | (0.207) | (0.207) | (0.206) | (0.207) | (0.207) |
| *Geographical location (ref. group = Outer regional or remote)* | | | | | |
| Major city | -0.091 | -0.093 | -0.097 | -0.092 | -0.092 |
| | (0.287) | (0.286) | (0.286) | (0.286) | (0.286) |
| Inner regional | -0.175 | -0.178 | -0.187 | -0.177 | -0.177 |
| | (0.286) | (0.286) | (0.285) | (0.286) | (0.286) |
| *State (ref. group = Victoria (VIC))* | | | | | |
| New South Wales (NSW) | -0.702 | -0.697 | -0.708 | -0.698 | -0.698 |
| | (0.427) | (0.427) | (0.427) | (0.427) | (0.427) |
| Queensland (QLD) | -0.730 | -0.732 | -0.733 | -0.732 | -0.732 |
| | (0.427) | (0.427) | (0.426) | (0.427) | (0.427) |
| South Australia (SA) | -0.784 | -0.786 | -0.774 | -0.789 | -0.789 |
| | (0.498) | (0.502) | (0.496) | (0.501) | (0.501) |
| Western Australia (WA) | -1.003 | -0.996 | -1.023 | -0.997 | -0.997 |
| | (0.544) | (0.550) | (0.541) | (0.549) | (0.549) |
| Tasmania (TAS) | -0.549 | -0.540 | -0.570 | -0.546 | -0.546 |
| | (0.561) | (0.562) | (0.561) | (0.562) | (0.562) |
| Northern Territory (NT) | -1.563 | -1.557 | -1.597 | -1.557 | -1.557 |
| | (1.190) | (1.191) | (1.185) | (1.191) | (1.192) |
| Australian Capital Territory (ACT) | -0.443 | -0.433 | -0.474 | -0.431 | -0.431 |
| | (0.644) | (0.646) | (0.643) | (0.646) | (0.646) |
| *Interview characteristics* | | | | | |
| Other adults present during i'view | 0.009 | 0.009 | 0.007 | 0.009 | 0.009 |
| | (0.047) | (0.047) | (0.047) | (0.047) | (0.047) |
| Interviewed by phone | 0.031 | 0.029 | 0.029 | 0.032 | 0.032 |
| | (0.053) | (0.054) | (0.054) | (0.054) | (0.054) |
| Constant | 7.904*** | 7.924*** | 7.953*** | 7.926*** | 7.926*** |
| | (0.829) | (0.827) | (0.821) | (0.828) | (0.827) |
| R-squared | 0.053 | 0.053 | 0.054 | 0.052 | 0.052 |
| Rho | 0.611 | 0.610 | 0.611 | 0.610 | 0.610 |
| Observations | 7594 | 7594 | 7594 | 7594 | 7594 |

*Notes*: WFH = Working from home.
**p*<0.05, ***p*<0.01, ****p*<0.001.

*Table A.6.* The Impact of Children on the Relationship Between Working from Home and Job Satisfaction (Fixed Effects Regression Results)

| | *Female* | | *Male* | |
|---|---|---|---|---|
| Variable | *Without children* | *With children* | *Without children* | *With children* |
| *Share of hours working from home (%) (ref. group = 0)* | | | | |
| 1-19 | 0.016 | 0.220* | -0.043 | 0.234** |
| | (0.128) | (0.110) | (0.108) | (0.084) |
| 20-39 | 0.143 | 0.210 | -0.251 | -0.052 |
| | (0.165) | (0.130) | (0.159) | (0.111) |
| 40-59 | 0.207 | 0.230 | 0.069 | 0.247 |
| | (0.194) | (0.192) | (0.190) | (0.147) |
| 60-79 | 0.231 | 0.940*** | 0.137 | 0.342* |
| | (0.270) | (0.246) | (0.255) | (0.166) |
| 80-99 | 0.265 | 0.569** | -0.010 | 0.225 |
| | (0.289) | (0.216) | (0.331) | (0.265) |
| 100 | 0.235 | 0.160 | 0.004 | 0.109 |
| | (0.126) | (0.107) | (0.115) | (0.119) |
| *Year = 2021* | 0.051 | -0.103 | 0.139* | 0.104 |
| | (0.075) | (0.079) | (0.069) | (0.068) |
| *Age group (ref. group = 35-44* years*)* | | | | |
| 15-24 years | 0.164 | -1.463** | -0.096 | -0.182 |
| | (0.292) | (0.471) | (0.268) | (0.363) |
| 25-34 years | 0.137 | -0.368* | -0.109 | -0.030 |
| | (0.222) | (0.150) | (0.199) | (0.129) |
| 45-54 years | -0.248 | 0.228 | -0.060 | 0.061 |
| | (0.187) | (0.147) | (0.205) | (0.147) |
| 55-64 years | -0.436 | 0.160 | -0.128 | -0.120 |
| | (0.326) | (0.222) | (0.313) | (0.263) |
| 65 years or older | -0.272 | 1.012* | -0.055 | -0.220 |
| | (0.388) | (0.508) | (0.415) | (0.445) |
| *Marital / partnering status (ref. group= Single)* | | | | |
| Married | 0.178 | -0.194 | -0.233 | 0.513 |
| | (0.224) | (0.260) | (0.179) | (0.355) |
| Cohabiting | 0.028 | -0.004 | 0.068 | 0.224 |
| | (0.149) | (0.244) | (0.140) | (0.368) |
| *Health status* | | | | |
| Has restrictive long-term health condition or disability | -0.011 | -0.149 | -0.037 | 0.028 |
| | (0.155) | (0.156) | (0.132) | (0.138) |
| *Educational attainment (ref. group = Year 11 and below)* | | | | |
| Year 12 | 0.292 | 0.496 | 0.295 | 0.206 |
| | (1.064) | (1.089) | (0.822) | (0.762) |
| Vocational qualification or diploma | 0.550 | 1.860* | 0.361 | -0.079 |
| | (0.955) | (0.822) | (0.659) | (0.585) |
| Bachelor's degree and higher | 1.313 | 2.419* | 0.188 | -0.145 |
| | (1.115) | (1.055) | (0.868) | (0.714) |
| *Employment type (ref. group = Permanent employee)* | | | | |
| Fixed-term contract employee | 0.164 | 0.145 | -0.109 | -0.283 |
| | (0.135) | (0.149) | (0.119) | (0.152) |
| Casual employee | 0.059 | 0.015 | -0.192 | -0.178 |
| | (0.173) | (0.167) | (0.148) | (0.208) |
| Self-employed | 0.107 | 0.173 | 0.062 | 0.055 |
| | (0.251) | (0.225) | (0.187) | (0.159) |
| Others | 0.600 | 0.619 | 0.081 | 1.074 |
| | (0.521) | (0.469) | (0.316) | (0.705) |

| | *Female* | | *Male* | |
|---|---|---|---|---|
| Variable | *Without children* | *With children* | *Without children* | *With children* |
| *Tenure with current employer (ref. group = <1 year)* | | | | |
| 1 year to less than 2 years | -0.143 | 0.129 | -0.245 | 0.043 |
| | (0.155) | (0.157) | (0.139) | (0.136) |
| 2 to < 5 years | -0.348** | -0.092 | -0.263** | -0.154 |
| | (0.111) | (0.107) | (0.100) | (0.101) |
| 5 to < 10 years | -0.505*** | -0.281* | -0.612*** | -0.442*** |
| | (0.147) | (0.125) | (0.126) | (0.123) |
| 10 to < 20 years | -0.422* | -0.243 | -0.644*** | -0.543*** |
| | (0.210) | (0.149) | (0.171) | (0.141) |
| 20 or more years | -0.494* | -0.140 | -0.636** | -0.590** |
| | (0.235) | (0.196) | (0.216) | (0.213) |
| *Occupation (ref. group = Labourers)* | | | | |
| Managers | 0.206 | -0.291 | 0.464* | -0.244 |
| | (0.317) | (0.374) | (0.219) | (0.217) |
| Professionals | 0.424 | -0.059 | 0.559* | -0.303 |
| | (0.309) | (0.372) | (0.247) | (0.237) |
| Technicians & trades workers | 0.660 | 0.130 | 0.252 | -0.093 |
| | (0.407) | (0.419) | (0.217) | (0.239) |
| Community & personal service workers | 0.245 | -0.383 | 0.737* | 0.469 |
| | (0.319) | (0.370) | (0.344) | (0.402) |
| Clerical & administrative workers | 0.264 | -0.005 | 0.183 | -0.002 |
| | (0.307) | (0.381) | (0.262) | (0.290) |
| Sales workers | 0.269 | -0.429 | 0.189 | 0.039 |
| | (0.346) | (0.470) | (0.274) | (0.303) |
| Machinery operators & drivers | -0.030 | -0.703 | -0.165 | 0.263 |
| | (0.350) | (0.801) | (0.236) | (0.240) |
| *Other job characteristics* | | | | |
| Usual hours worked per week in all jobs | 0.016 | 0.034* | 0.015 | 0.031 |
| | (0.014) | (0.014) | (0.015) | (0.018) |
| Usual hours worked per week in all jobs (squared) | -0.000 | -0.000* | -0.000 | -0.000* |
| | (0.000) | (0.000) | (0.000) | (0.000) |
| Multiple job holder | -0.084 | 0.227 | -0.175 | 0.167 |
| | (0.150) | (0.166) | (0.164) | (0.163) |
| Normally supervise work of other employees | -0.171 | 0.024 | -0.124 | 0.078 |
| | (0.099) | (0.083) | (0.079) | (0.077) |
| Trade union member | -0.502** | -0.104 | -0.236 | -0.056 |
| | (0.164) | (0.170) | (0.173) | (0.164) |
| Public sector | 0.174 | -0.049 | 0.205 | -0.138 |
| | (0.173) | (0.143) | (0.250) | (0.199) |
| *Firm size (ref. group = Small (0-19 employees))* | | | | |
| Medium (20-99 employees) | -0.055 | -0.242 | -0.240 | 0.206 |
| | (0.229) | (0.175) | (0.138) | (0.156) |
| Large (100-499 employees) | -0.001 | -0.301 | -0.308 | 0.139 |
| | (0.207) | (0.202) | (0.165) | (0.176) |
| Very large (500 or more) | -0.185 | -0.326 | 0.035 | 0.104 |
| | (0.199) | (0.185) | (0.169) | (0.175) |
| Firm size unknown | -0.229 | -0.073 | -0.090 | 0.304 |
| | (0.225) | (0.218) | (0.238) | (0.228) |
| *Industry (ref. group = Professional, scientific & technical services)* | | | | |
| Agriculture, forestry & fishing | -0.445 | 0.217 | -0.425 | -0.511 |
| | (0.616) | (0.547) | (0.341) | (0.397) |
| Mining | 1.039 | -0.550 | -0.540 | 0.116 |
| | (0.786) | (0.380) | (0.453) | (0.279) |

| Variable | *Female* *Without children* | *Female* *With children* | *Male* *Without children* | *Male* *With children* |
|---|---|---|---|---|
| Manufacturing | 0.694 | -0.298 | -0.358 | -0.287 |
| | (0.549) | (0.376) | (0.267) | (0.216) |
| Electricity, gas, water & waste services | 0.017 | 1.264 | -0.446 | -0.249 |
| | (0.516) | (0.726) | (0.435) | (0.354) |
| Construction | 1.176 | -0.526 | -0.454* | -0.330 |
| | (0.640) | (0.481) | (0.216) | (0.259) |
| Wholesale trade | 0.290 | -0.296 | -0.612* | -0.082 |
| | (0.441) | (0.374) | (0.271) | (0.268) |
| Retail trade | 0.240 | 0.057 | -0.584 | -0.414 |
| | (0.406) | (0.323) | (0.334) | (0.327) |
| Accommodation & food services | 0.393 | -0.221 | -1.817*** | -0.787 |
| | (0.359) | (0.513) | (0.420) | (0.674) |
| Transport, postal & warehousing | 0.420 | -0.422 | -0.234 | -0.741* |
| | (0.431) | (0.490) | (0.267) | (0.329) |
| Information media & telecommunications | 0.570 | -0.158 | -0.004 | 0.935 |
| | (0.619) | (0.388) | (0.384) | (0.553) |
| Financial & insurance services | 0.040 | -0.151 | -0.250 | -0.103 |
| | (0.489) | (0.402) | (0.358) | (0.302) |
| Rental, hiring & real estate services | 0.382 | -0.411 | -0.360 | 0.405 |
| | (0.481) | (0.711) | (0.374) | (0.662) |
| Administrative & support services | 0.491 | -0.286 | -0.184 | 0.053 |
| | (0.327) | (0.385) | (0.305) | (0.305) |
| Public administration & safety | 0.568 | 0.712 | -0.378 | -0.254 |
| | (0.310) | (0.365) | (0.302) | (0.269) |
| Education & training | 0.403 | 0.312 | -0.087 | 0.496 |
| | (0.405) | (0.314) | (0.394) | (0.432) |
| Health care & social assistance | 0.428 | 0.587* | -0.556 | 0.033 |
| | (0.319) | (0.281) | (0.401) | (0.277) |
| Arts & recreation services | 0.881* | 0.653 | -0.597 | 0.684 |
| | (0.361) | (0.480) | (0.395) | (0.447) |
| Other services | 0.266 | 0.106 | -0.478 | -0.661* |
| | (0.420) | (0.304) | (0.327) | (0.277) |
| *Geographical location (ref. group = Outer regional or remote)* | | | | |
| Major city | -0.004 | -0.470 | -0.515 | -0.704 |
| | (0.368) | (0.473) | (0.438) | (0.426) |
| Inner regional | -0.059 | 0.184 | -0.729 | -0.297 |
| | (0.313) | (0.339) | (0.454) | (0.348) |
| *State (ref. group = Victoria (VIC)* | | | | |
| New South Wales (NSW) | 0.202 | 0.205 | -1.558** | 0.697 |
| | (0.475) | (0.385) | (0.554) | (0.451) |
| Queensland (QLD) | -0.013 | -0.590 | -1.513** | 1.116* |
| | (0.445) | (0.416) | (0.519) | (0.517) |
| South Australia (SA) | 0.374 | 2.037** | -1.033 | -0.457 |
| | (0.606) | (0.664) | (0.658) | (1.224) |
| Western Australia (WA) | -0.157 | 2.330*** | -1.274* | -0.979 |
| | (1.005) | (0.540) | (0.601) | (0.877) |
| Tasmania (TAS) | -0.032 | | -1.358* | 1.285 |
| | (0.697) | | (0.627) | (0.681) |
| Northern Territory (NT) | | 3.503*** | -2.943 | -0.675 |
| | | (0.784) | (1.995) | (1.112) |
| Australian Capital Territory (ACT) | -0.280 | 0.669 | -0.593 | -0.673 |
| | (0.740) | (0.644) | (0.845) | (0.896) |

| Variable | *Female* | | *Male* | |
|---|---|---|---|---|
| | *Without children* | *With children* | *Without children* | *With children* |
| *Interview characteristics* | | | | |
| Other adults present during interview | -0.096 | -0.055 | 0.053 | 0.037 |
| | (0.095) | (0.077) | (0.077) | (0.063) |
| Interviewed by phone | 0.081 | 0.168 | -0.015 | 0.062 |
| | (0.089) | (0.087) | (0.080) | (0.080) |
| Constant | 6.592*** | 5.619*** | 9.581*** | 7.298*** |
| | (1.105) | (1.044) | (0.880) | (0.927) |
| R-squared | 0.062 | 0.104 | 0.083 | 0.090 |
| Rho | 0.619 | 0.745 | 0.652 | 0.691 |
| Observations | 3480 | 3660 | 3802 | 3792 |

*Notes*: *$p$<0.05, **$p$<0.01, ***$p$<0.001.

*Table A.7*. The Impact of Living in a Lockdown State on the Relationship Between Working from Home and Job Satisfaction (Fixed Effects Regression Results)

| | *Female* | | *Male* | |
|---|---|---|---|---|
| Variable | *Lockdown states (NSW + VIC)* | *Other states* | *Lockdown states (NSW + VIC)* | *Other states* |
| *Share of hours working from home (%) (ref. group = 0)* | | | | |
| 1-19 | 0.186 | 0.031 | 0.038 | 0.144 |
| | (0.098) | (0.133) | (0.088) | (0.092) |
| 20-39 | 0.331** | 0.012 | -0.114 | -0.067 |
| | (0.113) | (0.161) | (0.116) | (0.134) |
| 40-59 | 0.198 | 0.464* | 0.161 | 0.160 |
| | (0.163) | (0.226) | (0.142) | (0.173) |
| 60-79 | 0.726** | 0.485 | 0.064 | 0.474* |
| | (0.228) | (0.280) | (0.157) | (0.219) |
| 80-99 | 0.302 | 0.661* | 0.090 | 0.022 |
| | (0.195) | (0.260) | (0.212) | (0.312) |
| 100 | 0.168 | 0.387* | -0.009 | 0.247 |
| | (0.086) | (0.191) | (0.095) | (0.156) |
| *Year = 2021* | 0.092 | -0.037 | -0.067 | 0.131** |
| | (0.116) | (0.062) | (0.119) | (0.050) |
| *Age group (ref. group = 35-44* years*)* | | | | |
| 15-24 years | -0.183 | -0.259 | -0.070 | -0.038 |
| | (0.268) | (0.316) | (0.261) | (0.262) |
| 25-34 years | -0.061 | -0.322 | -0.088 | 0.034 |
| | (0.155) | (0.195) | (0.142) | (0.142) |
| 45-54 years | 0.288 | -0.127 | -0.098 | 0.265 |
| | (0.186) | (0.164) | (0.156) | (0.182) |
| 55-64 years | 0.198 | -0.393 | -0.234 | 0.048 |
| | (0.250) | (0.295) | (0.287) | (0.249) |
| 65 years or older | 0.429 | -0.080 | -0.388 | 0.157 |
| | (0.354) | (0.378) | (0.383) | (0.415) |
| *Marital / partnering status (ref. group= Single)* | | | | |
| Married | 0.055 | -0.258 | -0.063 | 0.138 |
| | (0.211) | (0.264) | (0.213) | (0.186) |
| Cohabiting | 0.0310 | -0.274 | 0.100 | 0.100 |
| | (0.171) | (0.205) | (0.163) | (0.157) |
| *Age of youngest child interacted with dependence (ref. group = No children)* | | | | |
| Aged 0 to 4 years | 0.252 | 0.014 | -0.138 | -0.058 |
| | (0.167) | (0.232) | (0.129) | (0.154) |
| Aged 5 to 14 years | 0.346 | 0.176 | -0.035 | 0.293 |
| | (0.183) | (0.243) | (0.158) | (0.155) |
| Dependent child aged 15 to 24 | 0.138 | 0.062 | 0.006 | 0.233 |
| | (0.153) | (0.228) | (0.187) | (0.164) |
| Independent child | -0.092 | -0.164 | 0.191 | 0.036 |
| | (0.143) | (0.215) | (0.153) | (0.177) |
| *Health status* | | | | |
| Has restrictive long-term health condition or disability | -0.106 | -0.012 | -0.059 | -0.052 |
| | (0.120) | (0.186) | (0.134) | (0.132) |
| *Educational attainment (ref. group = Year 11 and below)* | | | | |
| Year 12 | 1.139 | -0.834 | 1.149 | 0.753 |
| | (0.982) | (0.806) | (0.890) | (1.266) |
| Vocational qualification or diploma | 2.217** | -0.615 | 1.536** | 0.945 |
| | (0.795) | (0.615) | (0.579) | (1.264) |

| Variable | *Female*<br>*Lockdown states (NSW + VIC)* | *Female*<br>*Other states* | *Male*<br>*Lockdown states (NSW + VIC)* | *Male*<br>*Other states* |
|---|---|---|---|---|
| Bachelor's degree and higher | 2.347* | 0.227 | 1.284 | 0.336 |
| | (0.997) | (0.829) | (0.877) | (1.071) |
| *Employment type (ref. group = Permanent employee)* | | | | |
| Fixed-term contract employee | 0.079 | 0.171 | -0.153 | -0.294* |
| | (0.118) | (0.159) | (0.122) | (0.136) |
| Casual employee | -0.173 | 0.098 | 0.059 | -0.254 |
| | (0.156) | (0.178) | (0.177) | (0.152) |
| Self-employed | -0.069 | 0.379 | 0.166 | -0.048 |
| | (0.237) | (0.221) | (0.158) | (0.172) |
| Others | 1.460** | 0.211 | 0.313 | 0.590 |
| | (0.456) | (0.439) | (0.378) | (0.487) |
| *Tenure with current employer (ref. group = <1 year)* | | | | |
| 1 year to less than 2 years | -0.001 | -0.021 | -0.106 | -0.074 |
| | (0.134) | (0.175) | (0.137) | (0.128) |
| 2 to < 5 years | -0.234* | -0.202 | -0.249** | -0.188* |
| | (0.097) | (0.125) | (0.095) | (0.092) |
| 5 to < 10 years | -0.284* | -0.498*** | -0.505*** | -0.526*** |
| | (0.127) | (0.139) | (0.114) | (0.122) |
| 10 to < 20 years | -0.460** | -0.232 | -0.398** | -0.798*** |
| | (0.157) | (0.174) | (0.132) | (0.165) |
| 20 or more years | -0.466* | -0.243 | -0.744*** | -0.693*** |
| | (0.216) | (0.207) | (0.204) | (0.188) |
| *Occupation (ref. group = Labourers)* | | | | |
| Managers | -0.472 | 0.408 | 0.051 | 0.162 |
| | (0.373) | (0.328) | (0.213) | (0.203) |
| Professionals | -0.137 | 0.423 | 0.112 | 0.149 |
| | (0.374) | (0.319) | (0.221) | (0.243) |
| Technicians & trades workers | 0.410 | 0.210 | 0.190 | 0.019 |
| | (0.504) | (0.330) | (0.220) | (0.215) |
| Community & personal service workers | -0.400 | 0.164 | 0.247 | 0.901* |
| | (0.394) | (0.309) | (0.331) | (0.390) |
| Clerical & administrative workers | -0.227 | 0.471 | 0.067 | 0.236 |
| | (0.373) | (0.329) | (0.270) | (0.271) |
| Sales workers | -0.355 | 0.303 | 0.066 | 0.241 |
| | (0.400) | (0.400) | (0.277) | (0.311) |
| Machinery operators & drivers | 0.107 | -0.116 | 0.382 | -0.105 |
| | (0.545) | (0.352) | (0.250) | (0.203) |
| *Other job characteristics* | | | | |
| Usual hours worked per week in all jobs | 0.023 | 0.018 | 0.052** | -0.011 |
| | (0.012) | (0.014) | (0.016) | (0.015) |
| Usual hours worked per week in all jobs (squared) | -0.000* | -0.000 | -0.000*** | 0.000 |
| | (0.000) | (0.000) | (0.000) | (0.000) |
| Multiple job holder | 0.0059 | -0.090 | -0.089 | -0.023 |
| | (0.126) | (0.180) | (0.145) | (0.165) |
| Normally supervise work of other employees | -0.093 | -0.042 | -0.147* | 0.056 |
| | (0.084) | (0.099) | (0.068) | (0.080) |
| Trade union member | -0.145 | -0.427* | -0.122 | -0.216 |
| | (0.154) | (0.178) | (0.170) | (0.149) |
| Public sector | -0.169 | 0.402* | -0.329 | 0.223 |
| | (0.138) | (0.182) | (0.215) | (0.227) |

| Variable | Female: Lockdown states (NSW + VIC) | Female: Other states | Male: Lockdown states (NSW + VIC) | Male: Other states |
|---|---|---|---|---|
| *Firm size (ref. group = Small (0-19 employees))* | | | | |
| Medium (20-99 employees) | -0.019 | -0.288 | 0.031 | -0.133 |
| | (0.197) | (0.197) | (0.132) | (0.140) |
| Large (100-499 employees) | 0.046 | -0.313 | -0.017 | -0.151 |
| | (0.194) | (0.204) | (0.163) | (0.160) |
| Very large (500 or more) | -0.030 | -0.423* | 0.279 | -0.155 |
| | (0.195) | (0.196) | (0.165) | (0.159) |
| Firm size unknown | 0.121 | -0.291 | 0.361 | -0.185 |
| | (0.215) | (0.234) | (0.252) | (0.197) |
| *Industry (ref. group = Professional, scientific & technical services)* | | | | |
| Agriculture, forestry & fishing | 0.184 | -0.315 | -0.753** | -0.253 |
| | (0.742) | (0.384) | (0.279) | (0.340) |
| Mining | -0.615 | 0.101 | -0.435 | 0.522 |
| | (0.663) | (0.756) | (0.437) | (0.298) |
| Manufacturing | 0.300 | -0.582 | -0.432 | 0.108 |
| | (0.382) | (0.452) | (0.230) | (0.231) |
| Electricity, gas, water & waste services | 0.391 | 0.321 | -0.335 | -0.0838 |
| | (0.482) | (0.632) | (0.340) | (0.402) |
| Construction | 0.701 | -0.0797 | -0.415* | -0.164 |
| | (0.365) | (0.686) | (0.197) | (0.235) |
| Wholesale trade | -0.167 | -0.120 | -0.647** | 0.083 |
| | (0.407) | (0.375) | (0.238) | (0.269) |
| Retail trade | -0.218 | 0.394 | -0.457 | -0.268 |
| | (0.297) | (0.447) | (0.284) | (0.385) |
| Accommodation & food services | 0.287 | -0.223 | -1.470*** | -1.333* |
| | (0.407) | (0.434) | (0.442) | (0.520) |
| Transport, postal & warehousing | 0.035 | -0.073 | -0.817* | -0.076 |
| | (0.466) | (0.384) | (0.324) | (0.248) |
| Information media & telecommunications | 1.258*** | -1.316* | 0.361 | 0.939 |
| | (0.318) | (0.658) | (0.433) | (0.598) |
| Financial & insurance services | 0.0917 | 0.0641 | -0.381 | 0.287 |
| | (0.333) | (0.580) | (0.266) | (0.561) |
| Rental, hiring & real estate services | -0.141 | -0.363 | -0.554 | 0.277 |
| | (0.583) | (0.462) | (0.377) | (0.583) |
| Administrative & support services | 0.0940 | -0.125 | -0.358 | 0.213 |
| | (0.304) | (0.384) | (0.259) | (0.347) |
| Public administration & safety | 0.870*** | 0.258 | -0.990** | 0.191 |
| | (0.261) | (0.396) | (0.323) | (0.253) |
| Education & training | 0.486 | 0.0318 | -0.113 | 0.830 |
| | (0.281) | (0.395) | (0.329) | (0.461) |
| Health care & social assistance | 0.644** | 0.423 | -0.150 | 0.052 |
| | (0.247) | (0.334) | (0.289) | (0.274) |
| Arts & recreation services | 0.701* | 1.111** | -0.452 | -0.027 |
| | (0.347) | (0.425) | (0.329) | (0.444) |
| Other services | 0.501 | -0.024 | -0.816** | -0.002 |
| | (0.322) | (0.374) | (0.296) | (0.287) |
| *Geographical location (ref. group = Outer regional or remote)* | | | | |
| Major city | -0.310 | 0.081 | -0.351 | -0.052 |
| | (0.399) | (0.411) | (0.438) | (0.434) |
| Inner regional | 0.022 | -0.125 | -0.533 | -0.438 |
| | (0.368) | (0.327) | (0.354) | (0.470) |

| Variable | *Female* | | *Male* | |
|---|---|---|---|---|
| | *Lockdown states (NSW + VIC)* | *Other states* | *Lockdown states (NSW + VIC)* | *Other states* |
| *State (ref. group = Victoria (VIC)* | | | | |
| New South Wales (NSW) | 0.081 | | -0.657 | |
| | (0.492) | | (0.685) | |
| Queensland (QLD) | | -0.433 | | -0.065 |
| | | (0.508) | | (0.524) |
| South Australia (SA) | | -2.749** | | 0.434 |
| | | (1.041) | | (0.619) |
| Western Australia (WA) | | -0.153 | | -0.719 |
| | | (1.218) | | (0.560) |
| Tasmania (TAS) | | -1.514 | | 0.049 |
| | | (1.011) | | (0.686) |
| Northern Territory (NT) | | -0.763 | | -0.564 |
| | | (1.101) | | (1.281) |
| Australian Capital Territory (ACT) | | | | |
| *Interview characteristics* | | | | |
| Other adults present during interview | 0.081 | -0.153 | 0.048 | -0.025 |
| | (0.081) | (0.084) | (0.066) | (0.065) |
| Interviewed by phone | 0.061 | 0.106 | 0.234 | -0.054 |
| | (0.121) | (0.083) | (0.124) | (0.071) |
| Constant | 5.542*** | 8.855*** | 7.062*** | 7.792*** |
| | (1.005) | (1.121) | (0.907) | (1.345) |
| R-squared | 0.094 | 0.079 | 0.077 | 0.086 |
| Rho | 0.647 | 0.685 | 0.645 | 0.645 |
| Observations | 3839 | 3301 | 4118 | 3476 |

*Notes*: **p*<0.05, ***p*<0.01, ****p*<0.001.

*Table A.8.* The Impact of Working in a WFH Intensive Occupation on the Relationship Between Working from Home and Job Satisfaction (Fixed Effects Regression Results)

| Variable | *Female* WFH-intensive occs. | *Female* Other occs. | *Male* WFH-intensive occs. | *Male* Other occs. |
|---|---|---|---|---|
| *Share of hours working from home (%) (ref. group = 0)* | | | | |
| 1-19 | 0.250 | 0.009 | -0.039 | 0.185* |
| | (0.143) | (0.097) | (0.113) | (0.083) |
| 20-39 | 0.327* | 0.167 | -0.123 | -0.057 |
| | (0.156) | (0.124) | (0.126) | (0.143) |
| 40-59 | 0.265 | 0.216 | 0.023 | 0.287 |
| | (0.193) | (0.195) | (0.164) | (0.180) |
| 60-79 | 0.917*** | 0.395 | 0.151 | 0.142 |
| | (0.244) | (0.274) | (0.233) | (0.173) |
| 80-99 | 0.552* | -0.032 | 0.089 | -0.199 |
| | (0.224) | (0.225) | (0.273) | (0.261) |
| 100 | 0.061 | 0.274** | 0.002 | 0.208 |
| | (0.140) | (0.105) | (0.130) | (0.148) |
| *Year = 2021* | -0.142 | 0.037 | -0.055 | 0.152** |
| | (0.116) | (0.060) | (0.105) | (0.053) |
| *Age group (ref. group = 35-44* years*)* | | | | |
| 15-24 years | -0.503 | -0.199 | 0.019 | -0.186 |
| | (0.429) | (0.242) | (0.406) | (0.222) |
| 25-34 years | 0.173 | -0.268 | -0.048 | -0.134 |
| | (0.181) | (0.161) | (0.178) | (0.124) |
| 45-54 years | 0.182 | 0.122 | 0.153 | -0.030 |
| | (0.194) | (0.174) | (0.206) | (0.152) |
| 55-64 years | 0.451 | -0.123 | -0.032 | -0.259 |
| | (0.378) | (0.244) | (0.344) | (0.232) |
| 65 years or older | 0.888 | 0.031 | 0.200 | -0.574 |
| | (0.511) | (0.315) | (0.486) | (0.319) |
| *Marital / partnering status (ref. group= Single)* | | | | |
| Married | -0.187 | -0.128 | 0.697* | -0.121 |
| | (0.336) | (0.189) | (0.325) | (0.165) |
| Cohabiting | -0.289 | -0.009 | 0.264 | 0.109 |
| | (0.305) | (0.140) | (0.243) | (0.134) |
| *Age of youngest child interacted with dependence (ref. group = No children)* | | | | |
| Aged 0 to 4 years | 0.617** | -0.120 | -0.406* | -0.148 |
| | (0.234) | (0.176) | (0.176) | (0.129) |
| Aged 5 to 14 years | 0.797** | 0.086 | -0.405 | 0.120 |
| | (0.263) | (0.182) | (0.221) | (0.134) |
| Dependent child aged 15 to 24 | 0.505* | -0.054 | -0.146 | 0.113 |
| | (0.243) | (0.158) | (0.251) | (0.149) |
| Independent child | 0.001 | -0.191 | 0.023 | 0.094 |
| | (0.296) | (0.140) | (0.311) | (0.127) |
| *Health status* | | | | |
| Has restrictive long-term health condition or disability | 0.056 | -0.102 | 0.387 | -0.142 |
| | (0.257) | (0.118) | (0.219) | (0.107) |
| *Educational attainment (ref. group = Year 11 and below)* | | | | |
| Year 12 | 2.303 | 0.343 | -0.276 | 0.272 |
| | (1.269) | (0.820) | (0.689) | (0.891) |
| Vocational qualification or diploma | 3.835*** | 0.387 | -0.542 | 0.844 |
| | (0.955) | (0.649) | (0.445) | (0.744) |

| Variable | Female: WFH-intensive occs. | Female: Other occs. | Male: WFH-intensive occs. | Male: Other occs. |
|---|---|---|---|---|
| Bachelor's degree and higher | 4.455*** | 1.446 | | 0.312 |
| | (1.213) | (0.910) | | (0.810) |
| *Employment type (ref. group = Permanent employee)* | | | | |
| Fixed-term contract employee | 0.101 | 0.223 | -0.432* | -0.162 |
| | (0.174) | (0.116) | (0.179) | (0.115) |
| Casual employee | -0.219 | 0.067 | 0.097 | -0.038 |
| | (0.292) | (0.133) | (0.390) | (0.128) |
| Self-employed | 0.215 | 0.258 | -0.183 | 0.221 |
| | (0.402) | (0.176) | (0.291) | (0.142) |
| Others | 0.200 | 1.253** | -0.045 | 0.647 |
| | (0.450) | (0.428) | (0.445) | (0.410) |
| *Tenure with current employer (ref. group = <1 year)* | | | | |
| 1 year to less than 2 years | 0.138 | 0.013 | -0.196 | -0.047 |
| | (0.215) | (0.126) | (0.186) | (0.114) |
| 2 to < 5 years | -0.357* | -0.216* | -0.396** | -0.173* |
| | (0.144) | (0.092) | (0.128) | (0.082) |
| 5 to < 10 years | -0.580** | -0.429*** | -0.632*** | -0.462*** |
| | (0.187) | (0.108) | (0.155) | (0.105) |
| 10 to < 20 years | -0.824** | -0.299* | -0.755*** | -0.483*** |
| | (0.260) | (0.129) | (0.186) | (0.128) |
| 20 or more years | -0.618 | -0.400* | -0.757** | -0.633*** |
| | (0.374) | (0.164) | (0.263) | (0.178) |
| *Occupation (ref. group = Labourers)* | | | | |
| Managers | -0.429 | 0.116 | -0.904 | 0.069 |
| | (0.605) | (0.275) | (0.540) | (0.176) |
| Professionals | -0.221 | 0.040 | -1.018 | 0.149 |
| | (0.600) | (0.287) | (0.541) | (0.241) |
| Technicians & trades workers | -1.432* | 0.399 | -1.029 | 0.163 |
| | (0.685) | (0.330) | (0.642) | (0.172) |
| Community & personal service workers | 0.245 | -0.075 | -2.699*** | 0.468 |
| | (0.849) | (0.268) | (0.752) | (0.293) |
| Clerical & administrative workers | -0.309 | 0.057 | -0.592 | 0.304 |
| | (0.557) | (0.279) | (0.517) | (0.233) |
| Sales workers | | -0.001 | | 0.108 |
| | | (0.306) | | (0.223) |
| Machinery operators & drivers | | -0.016 | | 0.192 |
| | | (0.332) | | (0.167) |
| *Other job characteristics* | | | | |
| Usual hours worked per week in all jobs | 0.028 | 0.012 | 0.006 | 0.018 |
| | (0.021) | (0.011) | (0.020) | (0.014) |
| Usual hours worked per week in all jobs (squared) | -0.001 | -0.000 | -0.000 | -0.000 |
| | (0.000) | (0.000) | (0.000) | (0.000) |
| Multiple job holder | 0.120 | -0.057 | -0.229 | 0.113 |
| | (0.288) | (0.122) | (0.225) | (0.142) |
| Normally supervise work of other employees | -0.085 | -0.011 | 0.054 | -0.072 |
| | (0.133) | (0.074) | (0.104) | (0.065) |
| Trade union member | -0.110 | -0.319* | -0.286 | -0.170 |
| | (0.352) | (0.124) | (0.180) | (0.130) |
| Public sector | 0.147 | -0.017 | 0.316 | 0.185 |
| | (0.271) | (0.123) | (0.195) | (0.191) |
| *Firm size (ref. group = Small (0-19 employees))* | | | | |
| Medium (20-99 employees) | 0.385 | -0.149 | -0.376 | 0.137 |
| | (0.316) | (0.144) | (0.215) | (0.116) |

| Variable | Female | | Male | |
|---|---|---|---|---|
| | *WFH-intensive occs.* | *Other occs.* | *WFH-intensive occs.* | *Other occs.* |
| Large (100-499 employees) | 0.546 | -0.193 | -0.398 | 0.109 |
| | (0.354) | (0.154) | (0.252) | (0.141) |
| Very large (500 or more) | 0.503 | -0.241 | -0.240 | 0.215 |
| | (0.390) | (0.148) | (0.260) | (0.139) |
| Firm size unknown | -0.045 | 0.006 | -0.018 | 0.234 |
| | (0.426) | (0.170) | (0.339) | (0.188) |
| *Industry (ref. group = Professional, scientific & technical services)* | | | | |
| Agriculture, forestry & fishing | 2.349*** | -0.724 | -0.801* | -0.508 |
| | (0.710) | (0.501) | (0.322) | (0.340) |
| Mining | -2.883*** | 0.461 | 0.985 | -0.234 |
| | (0.617) | (0.524) | (0.721) | (0.330) |
| Manufacturing | -0.130 | 0.067 | -0.479 | -0.194 |
| | (0.673) | (0.410) | (0.422) | (0.292) |
| Electricity, gas, water & waste services | 0.757 | 0.087 | -0.065 | -0.326 |
| | (1.216) | (0.621) | (0.579) | (0.389) |
| Construction | 0.447 | 0.376 | -0.685 | -0.332 |
| | (0.540) | (0.486) | (0.387) | (0.275) |
| Wholesale trade | 0.442 | -0.329 | -0.375 | -0.453 |
| | (0.836) | (0.368) | (0.441) | (0.303) |
| Retail trade | 0.319 | -0.262 | -0.147 | -0.630 |
| | (0.412) | (0.355) | (0.325) | (0.369) |
| Accommodation & food services | -1.249* | -0.090 | -1.149 | -1.415** |
| | (0.497) | (0.381) | (1.041) | (0.480) |
| Transport, postal & warehousing | 0.984 | -0.439 | -0.946 | -0.392 |
| | (0.544) | (0.393) | (0.768) | (0.302) |
| Information media & telecommunications | 0.838* | 0.132 | 0.083 | 0.079 |
| | (0.368) | (0.722) | (0.485) | (0.668) |
| Financial & insurance services | -0.163 | -0.553 | -0.020 | -1.122 |
| | (0.378) | (0.438) | (0.217) | (1.326) |
| Rental, hiring & real estate services | 0.741 | -0.411 | -0.604 | -0.218 |
| | (0.841) | (0.536) | (0.530) | (0.477) |
| Administrative & support services | 0.263 | -0.341 | -0.385 | 0.240 |
| | (0.359) | (0.376) | (0.341) | (0.339) |
| Public administration & safety | -0.205 | 0.673 | -0.628* | -0.205 |
| | (0.366) | (0.354) | (0.282) | (0.354) |
| Education & training | -0.342 | 0.325 | 0.297 | 0.327 |
| | (0.584) | (0.334) | (0.288) | (0.522) |
| Health care & social assistance | 0.224 | 0.378 | -0.899* | -0.157 |
| | (0.412) | (0.305) | (0.418) | (0.326) |
| Arts & recreation services | 0.833** | 0.684 | -1.345* | 0.270 |
| | (0.319) | (0.429) | (0.549) | (0.425) |
| Other services | 0.784 | -0.054 | 0.355 | -0.572 |
| | (0.424) | (0.370) | (0.412) | (0.328) |
| *Geographical location (ref. group = Outer regional or remote)* | | | | |
| Major city | 0.455 | 0.036 | 0.778 | -0.113 |
| | (0.484) | (0.317) | (0.611) | (0.358) |
| Inner regional | 0.023 | 0.078 | 0.781 | -0.299 |
| | (0.458) | (0.280) | (0.574) | (0.353) |
| *State (ref. group = Victoria (VIC)* | | | | |
| New South Wales (NSW) | -0.495 | 0.049 | -0.596 | -0.911 |
| | (0.744) | (0.418) | (0.680) | (0.602) |
| Queensland (QLD) | 0.952 | -0.774 | -0.891 | -1.023 |
| | (0.516) | (0.445) | (0.584) | (0.563) |

| Variable | Female | | Male | |
|---|---|---|---|---|
| | *WFH-intensive occs.* | *Other occs.* | *WFH-intensive occs.* | *Other occs.* |
| South Australia (SA) | | 0.225 | 0.613 | -1.709* |
| | | (0.619) | (0.394) | (0.854) |
| Western Australia (WA) | -0.044 | -0.008 | -1.156** | -1.735* |
| | (1.249) | (0.957) | (0.408) | (0.852) |
| Tasmania (TAS) | | -0.523 | 0.862 | -1.069 |
| | | (0.696) | (0.589) | (0.911) |
| Northern Territory (NT) | | 2.547*** | | -2.294 |
| | | (0.720) | | (1.289) |
| Australian Capital Territory (ACT) | -0.422 | -0.049 | 0.541 | -1.646 |
| | (0.870) | (0.994) | (0.916) | (0.847) |
| *Interview characteristics* | | | | |
| Other adults present during interview | 0.059 | -0.099 | -0.108 | 0.015 |
| | (0.121) | (0.066) | (0.089) | (0.058) |
| Interviewed by phone | 0.255* | 0.076 | 0.072 | -0.014 |
| | (0.129) | (0.068) | (0.122) | (0.063) |
| Constant | 3.067* | 7.331*** | 9.175*** | 8.400*** |
| | (1.271) | (0.868) | (1.090) | (0.967) |
| R-squared | 0.174 | 0.064 | 0.112 | 0.068 |
| Rho | 0.784 | 0.653 | 0.683 | 0.649 |
| Observations | 1809 | 5295 | 1944 | 5564 |

*Notes*: WFH = Working from home.
*$p<0.05$, **$p<0.01$, ***$p<0.001$.
